# ITGB1 and DDR activation as novel mediators in acquired resistance to osimertinib and MEK inhibitors in EGFR-mutant NSCLC

**DOI:** 10.1038/s41598-023-50568-5

**Published:** 2024-01-04

**Authors:** Caterina De Rosa, Viviana De Rosa, Concetta Tuccillo, Virginia Tirino, Luisa Amato, Federica Papaccio, Davide Ciardiello, Stefania Napolitano, Giulia Martini, Fortunato Ciardiello, Floriana Morgillo, Francesca Iommelli, Carminia Maria Della Corte

**Affiliations:** 1 Department of Precision Medicine, University of Campania Luigi Vanvitelli, Naples, Italy; 2 Institute of Biostructures and Bioimaging, National Research Council, Naples, Italy; 3 Department of Experimental Medicine, University of Campania Luigi Vanvitelli, Naples, Italy; 4 Department of Medicine, Surgery and Dentistry, Scuola Medica Salernitana”, University of Salerno, Baronissi, Italy; 5 Division of Gastrointestinal Medical Oncology and Neuroendocrine Tumors, European Institute of Oncology (IEO), IRCCS, Milan, Italy

**Keywords:** Cancer therapeutic resistance, Tumour biomarkers

## Abstract

Osimertinib is a third-generation tyrosine kinase inhibitor clinically approved for first-line treatment of EGFR-mutant non-small cell lung cancer (NSCLC) patients. Although an impressive drug response is initially observed, in most of tumors, resistance occurs after different time and an alternative therapeutic strategy to induce regression disease is currently lacking. The hyperactivation of MEK/MAPKs, is one the most common event identified in osimertinib-resistant (OR) NSCLC cells. However, in response to selective drug pressure, the occurrence of multiple mechanisms of resistance may contribute to treatment failure. In particular, the epithelial-to-mesenchymal transition (EMT) and the impaired DNA damage repair (DDR) pathways are recognized as additional cause of resistance in NSCLC thus promoting tumor progression. Here we showed that concurrent upregulation of ITGB1 and DDR family proteins may be associated with an increase of EMT pathways and linked to both osimertinib and MEK inhibitor resistance to cell death. Furthermore, this study demonstrated the existence of an interplay between ITGB1 and DDR and highlighted, for the first time, that combined treatment of MEK inhibitor with DDRi may be relevant to downregulate ITGB1 levels and increase cell death in OR NSCLC cells.

## Introduction

The use of third generation tyrosine kinase inhibitor (TKI) osimertinib, is currently approved as first line of treatment for patients with advanced non-small cell lung cancer (NSCLC) harbouring both activating and T790M mutation of epidermal growth factor receptor (EGFR) and a great clinical benefit was observed^1,2^. However, despite the impressive therapeutic response, in most of patients a poor prognosis continues to be assessed due to the occurring of resistance within 9–14 months of treatment^3^. In particular, it is reported that the prolonged and selective drug pressure inhibiting the tyrosine kinase activity of EGFR may cause epigenetic rearrangements in cancer cells promoting the amplification of oncogenic downstream signaling such as MEK/MAPK cascade along with the activation of the epithelial-to-mesenchymal transition (EMT)^4,5^. The EMT is another relevant mechanism of resistance that may develop after treatment with osimertinib and that may be related to changes in the composition of the extracellular matrix (ECM) that strongly contribute to the occurrence of such process^6^. In particular, communication between ECM and cells is mediated by integrins that, upon ligand binding, induce the formation of focal adhesion complexes in the cell membrane. These complexes recruit focal adhesion kinases (FAK) or integrin-linked kinase (ILK), which activate intracellular signaling pathways, including NF-κB, PI3K, Src and Ras-MAPK cascades, involved in the regulation of proliferation, motility and survival^7^. The integrin family consists of alpha (α) and beta (β) subtypes. Among 18 α- and 8 β-subunits that pair and incorporate into 24 different heterodimers, integrin β1, β3, αv and α5 play a major role in cancer metastasis and progression of cancer patients^8^. In particular, integrin β1 (ITGB1), that may heterodimerize with several α subunits, has been identified at high levels in invasive tumors with an EMT signature and resistant to EGFR TKIs^9–12^. Furthermore, it is demonstrated that when ITGB1 is overexpressed and overactivated in cancer, such as in lung, colorectal, pancreatic and breast carcinoma it is also associated with a poor prognosis^13–16^. The levels and activation of integrins may also depend on the type of cancer and the site of metastasis, and several studies in this regard have shown that high levels of α5β1 integrin in advanced lung cancer^17^ and in lymph-node positive NSCLC cells^18^. Moreover, it was also shown that integrin α5β1 may facilitate cancer cell invasion and enhance EGFR signaling^19^. As a transmembrane protein, integrins possess different domains, including extracellular, transmembrane and cytoplasmic domains, which determine their multiple functions. Upon binding to the extracellular matrix (ECM), integrins intracellular machinery can mediate complex signal transduction pathways that promotes cell migration and tumor progression^20^. Although both α and β integrin subunits contain cytoplasmic domains, it appears that the β integrin cytoplasmic domain is primarily required for cytoskeletal interactions^21^ and all of the information necessary for integrin localization to focal adhesions is present in the β-cytoplasmic domain^22^. Furthermore, it is reported that the activation of ITGB1 intracellular signaling is related to upregulation of both MAPKs pathway and EMT program^12,23,24^ and an emergent role of ITGB1 contributing to the modulation of DNA damage repair (DDR) by affecting cell cycle checkpoints, apoptosis and ATM/Chk2 signaling in several cancer cells^25,26^ is also described. DDR is a complex signaling pathway that detects DNA injury and mobilizes the downstream cascade of DNA reparative mechanisms^27^. Ataxia-telangiectasia-mutated (ATM) is the first sensor and acts promoting the recruitment of poly(ADP-ribose)polymerase 1 (PARP1) to produce poly(ADP-ribose) (PAR) polymers and amplify DNA damage signaling. ATM is immediately auto-phosphorylated and rapidly initiates a phosphorylation cascade that targets downstream effectors such as the histone H2A variant H2A.X. When phosphorylated at serine 139, H2A.X forms the γH2A.X mark of damaged chromatin, which acts as a platform for the recruitment of DNA repair proteins^28^. Some studies found that the capacity of DDR was compromised in osimertinib-resistant (OR) cells, evidenced by increased levels of γH2A.X^5^. In addition, inhibition or knockdown of DNA-dependent protein kinase (DNA-PK), a key kinase driving non-homologous end-joining (NHEJ), sensitized the resistant cells to osimertinib^5^. For the present study, we explored the role of MEK/MAPK and ITGB1 levels contributing to osimertinib resistance in EGFR mutant NSCLCs and we assessed whether such resistance may be related to the activation of DDR pathways. In this respect, we hypothesized the existence of an interplay between ITGB1 and DDR contributing to progression of OR NSCLC tumors.

## Results

### Effect of osimertinib on cell viability and cell migration in parental and resistant NSCLC cell lines

**Figure 1:**
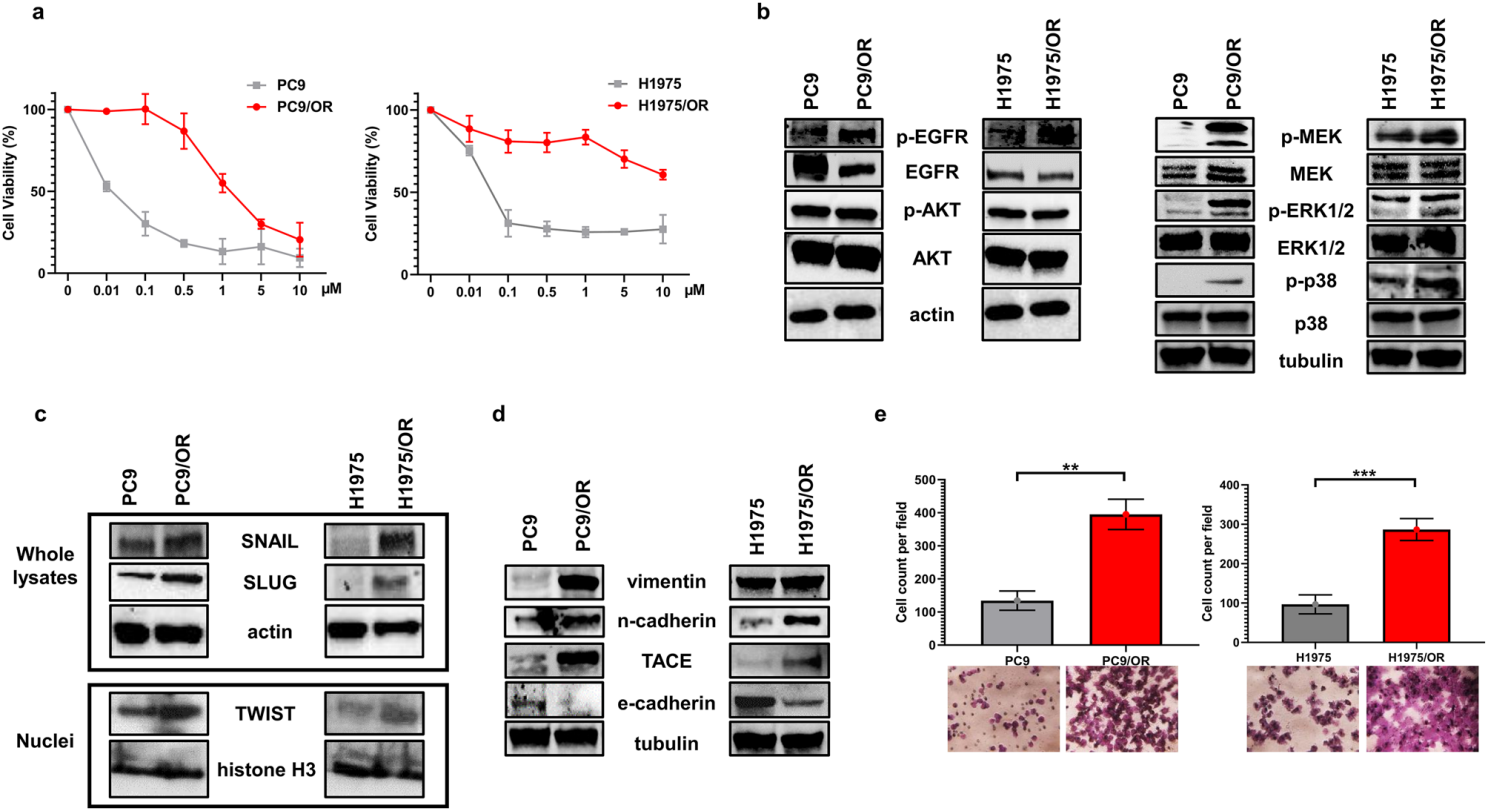
Hyperactivation of MAPK signaling along with EMT pathways in OR NSCLC cell lines. (**a**) Cell toxicity assay in parental (PC9, H1975) and resistant (PC9/OR, H1975/OR) cells exposed to increasing concentrations of osimertinib for 72 h. (**b**–**d**) Representative western blotting of whole cell lysates showing levels of phosphorylated and total forms of (**b**) EGFR, AKT, MEK, ERK1/2, p38, (**c**) expression of SNAIL and SLUG in whole cell lysates and TWIST in nuclear extracts and (**d**) levels of vimentin, n-cadherin, TACE and e-cadherin in both parental and OR NSCLC cell lines. Actin, tubulin and histone H3 were used to ensure equal loading. At least three independent experiments were performed. (**e**) Representative microscopic images and quantitative analysis of migration assay. Migrated cells (to 5% FBS) were stained with crystal violet (20× magnification) and counted. A mean of migrated cells per visual field of 3 replicate wells was graphically reported. Statistical significance **p < 0.01 and ***p < 0.001. At least three independent experiments were performed.

The sensitivity of the PC9, H1975, PC9/OR and H1975/OR cell lines to increasing concentrations of osimertinib was preliminarily tested by MTS assay and the results are shown in Fig. 1a. As expected, PC9 and H1975 cell lines were highly sensitive to osimertinib showing an IC50 of 0.0091 µM and 0.0159 µM, respectively. Conversely, a weak reduction of cell viability of PC9/OR and H1975/OR was observed thus confirming their resistance to osimertinib (IC50 = 4.8 µM and 14.7 µM, respectively). Furthermore, a strong increase of p-EGFR, p-MEK, p-ERK1/2 and p-p38 levels was observed in PC9/OR and H1975/OR cells compared to corresponding parental cells (Fig. 1b). Also, phosphorylated and total levels of AKT did not change between parental and resistant cell lines. These findings confirm a selective hyperactivation of MAPK signaling in these OR cellular models as a consequence of osimertinib selective pressure. Parallel experiments were also performed to investigate the modulation of EMT markers in response to EGFR inhibitor. In this respect, Fig. 1c showed a strong upregulation of transcriptional regulators SNAIL and SLUG in whole cell lysates and TWIST in nuclear extracts of OR cells compared to parental ones (Fig. 1c). These transcriptional factors correlate with increased metastatic properties and chromosomal instability^29^. To further confirm if the activation of these transcription factors relies on the activation of EMT, we investigated the expression levels of other EMT protein markers. In particular, a decrease of e-cadherin and an increase of TACE (TNF-α converting enzyme, also known as ADAM17), vimentin and n-cadherin were observed in OR cells compared to parental ones (Fig. 1d). The detection of high levels of such proteins were associated in PC9/OR and H1975/OR cells with a more aggressive and invasive phenotype then parental cells, as also confirmed by migration assays (*p* = 0.0011 and *p* = 0.0008, respectively). As showed in representative images of Fig. 1e, the highest number of migrated cells was observed in OR cells.

### Levels of ITGB1 and its downstream signaling activation

**Figure 2:**
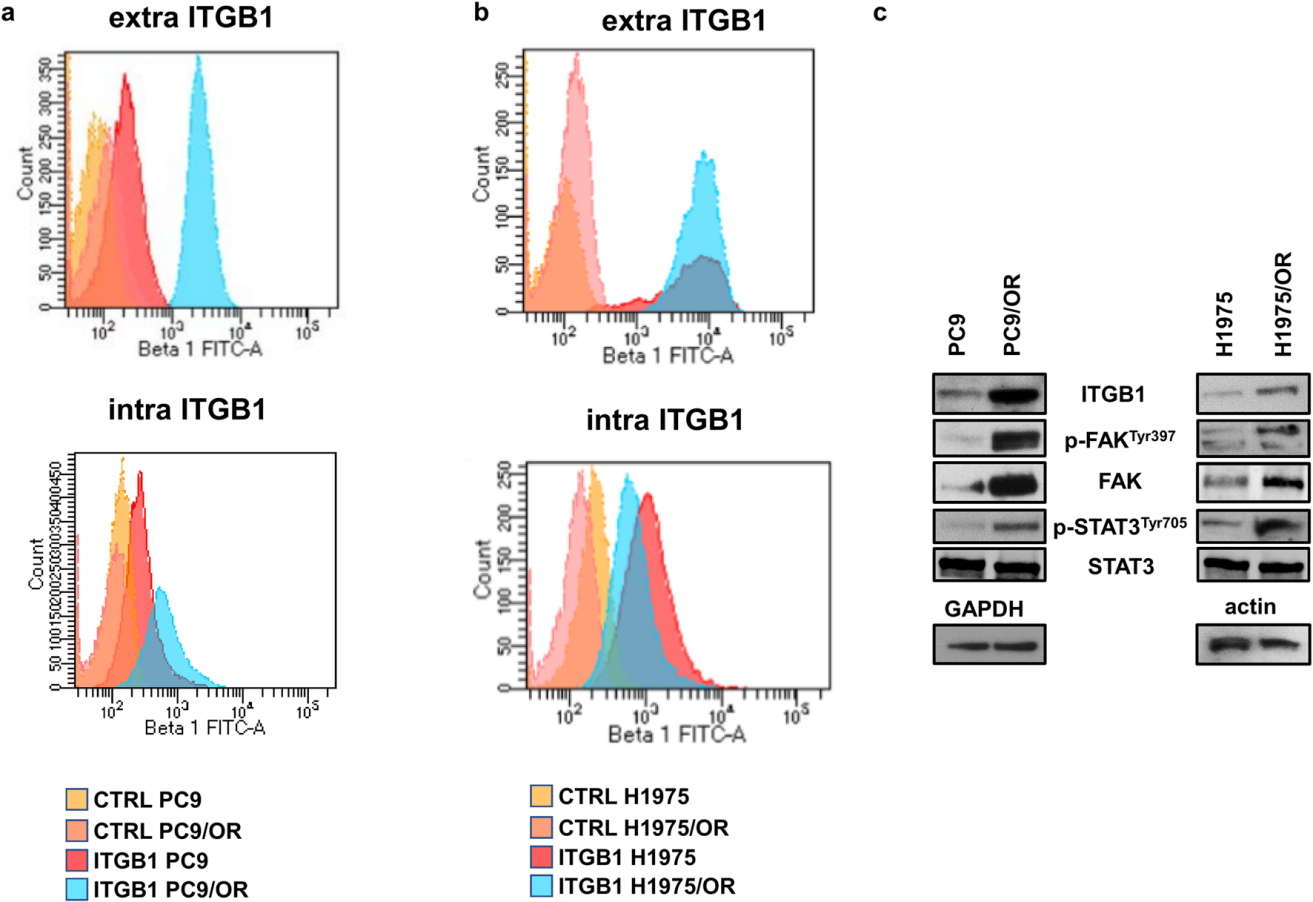
Levels of ITGB1 integrin and its downstream signaling activation in parental and OR NSCLC cells. (**a**, **b**) FACS analysis of extra and intracellular levels of ITGB1 in (**a**) PC9, PC9/OR, (**b**) H1975 and H1975/OR cells. (**c**) Western blotting of ITGB1 and its downstream signaling mediators detected in whole cell lysates. GAPDH or actin were used to ensure equal loading. At least three independent experiments were performed.

Since integrins play a key role in mediating adhesion, migration and EMT in cancer cells, we tested their levels and activation in parental and OR cells. In particular, we focused on integrin β1 (ITGB1) since it has been proved to be overexpressed during EMT activation and able to modulate EGFR signaling in EGFR-driven NSCLC cells. In this respect, Morello V. et al. demonstrated that EGFR is phosphorylated by ITGB1 even in the absence of EGF and that such integrin overexpression may be relevant for cell invasion^30^. Here, we hypothesized that the concurrent upregulation of MAPKs and EMT markers in OR cells may be linked to ITGB1 downstream signaling pathway activation. In this respect, flow cytometry (Fig. 2a) showed an increase of α5β1 levels on plasma membrane of PC9/OR cell population in comparison with PC9 cells (99.4% of positive cells vs 65.2% respectively). Whereas, for H1975 and H1975/OR cells we found that α5β1 is highly expressed and in equal levels in both cell populations (99% of positive cells). However, we also analyzed the mean fluorescence intensity (MFI) of α5β1 registered during FACS analysis and interestingly, we found a reduction of MFI for H1975/OR cell line (Supplementary Figure S1a) that may be also due to a β1 internalization. In this respect, we performed flow cytometry also to test intracellular levels of β1 and found that both extra- and intra-cellular β1 subunit were significantly increased only in PC9/OR cells (Fig. 2a, b). However, for both resistant cell lines we supposed that the distribution of extra- and intra-cellular β1 may vary in a context of potential subclones heterogeneity and under treatment pressure and hypothesized that along with high levels of ITGB1 also the integrin signaling overactivation may represent a marker of osimertinib resistance. In order to address this point, the activation of integrin pathway was also assessed by western blot analysis of ITGB1 levels along with p-FAK^Tyr397^, FAK, p-STAT3^Tyr705^ and STAT3 in all selected cell lines. As expected, immunoblot analysis showed ITGB1 overexpression in both PC9/OR and H1975/OR cells as well as an increase of p-FAK^Tyr397^ and p-STAT3^Tyr705^ (Fig. 2c) thus indicating a hyperactivation of ITGB1 signaling in two OR cell lines. These results are in agreement with several evidence demonstrating that FAK protein is an integrin pathway mediator also known as an upstream regulator of the STAT and MAPK signaling. In addition, with a second set of experiments, we assessed if high levels of total β1 integrin are mainly associated also with αv expression in OR resistant cells. αv integrin has been linked with tumor progression^31^ and its concurrent upregulation with β1 may contribute to promote cell migration. In particular, we found that levels of both extra- and intra-cellular αv subunit were higher in two OR cell lines then parental cells (Supplementary Figure S1b). However, the present study is mainly focused on β1 signaling overactivation as a marker of osimertinib resistance and regardless of which α subunit such integrin may be associated. More extensive experiments will need to better elucidate this aspect and may be the object of future studies.

### Effect of selumetinib on cell viability, MAPKs activation and levels of ITGB1

**Figure 3:**
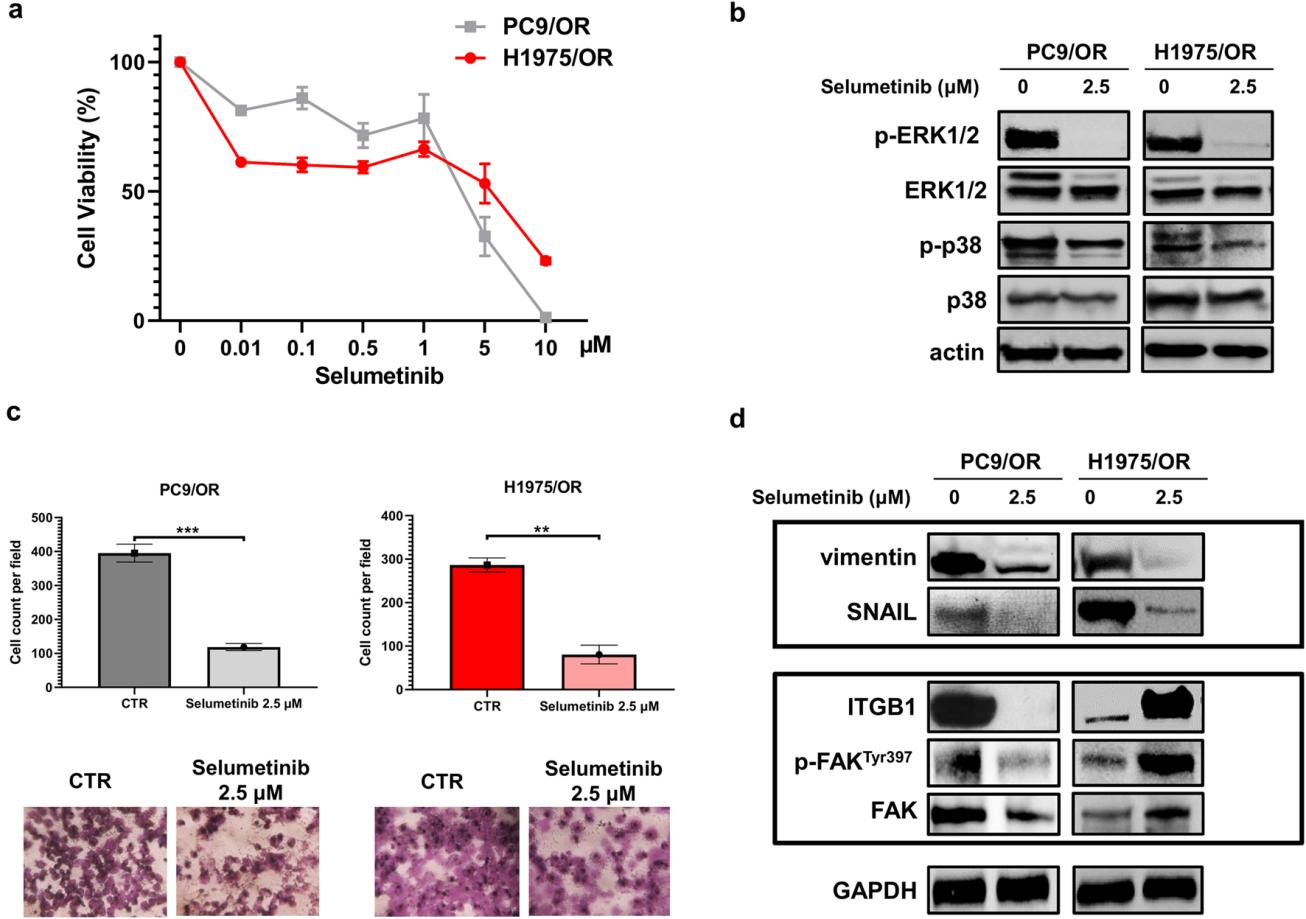
Effect of selumetinib on cell viability and migration. (**a**) MTS assay in PC9/OR and H1975/OR cells exposed to increasing concentrations of selumetinib for 72 h. Data are expressed as mean of three independent experiments ± SD. (**b**) Representative western blotting of whole cell lysates showing levels of phosphorylated and total forms of MAPKs (p-ERK1/2, ERK1/2, p-p38, p38). Actin was used to ensure equal loading. (**c**) Representative images (20× magnification) and quantitative analysis of untreated and treated cells migrated through the trans-well insert and stained with crystal violet. Statistical significance **p < 0.01 and ***p < 0.001. (**d**) Expression levels of EMT markers (vimentin and SNAIL), ITGB1, p-FAK^Tyr397^ and FAK in response to treatment with selumetinib (2.5 µM) for 72 h. GAPDH was used as equal loading.

Since our findings showed that the upregulation of MAPKs and ITGB1 signaling could represent a common mechanism of resistance in both PC9/OR and H1975/OR cells, we selected an inhibitor of MAPK signaling to test its effect on cell viability, cell migration, levels of p-ERK1/2/ERK, p-p38/p38 and EMT markers. In particular, we used selumetinib (AZD6244), a small molecule kinase inhibitor approved for clinical trials in refractory NSCLC patients^32^. Figure 3a showed that this inhibitor weakly reduced cell viability in both parental PC9 and H1975 (Supplementary Figure S2) and OR cell lines as shown by IC50 values (3.99 µM and 3.80 µM, respectively) whereas it strongly reduced MAPKs signaling pathways (Fig. 3b). In addition, selumetinib treatment was able to significantly reduce cell migration ability of PC9/OR (*p* = 0.0006) and H1975/OR (*p* = 0.0015) cells (Fig. 3c). Parallel experiments were performed to analyse whole cell lysates from untreated and treated cells and results showed that treatment with 2.5 μM selumetinib for 72 h in PC9/OR and H1975/OR cells was able to cause an impressive reduction of the EMT markers such as vimentin and SNAIL (Fig. 3d, upper panel). These findings are consistent with several studies demonstrating a relevant role of ERK1/2 and p38 MAPK in regulating EMT signaling^33,34^. In addition, a strong decrease of ITGB1 levels and its downstream signaling pathway (p-FAK^Tyr397^, FAK) was also observed in PC9/OR. Conversely, the same drug was able to induce a significant upregulation of ITGB1 levels and activation of its signaling mediators in H1975/OR (Fig. 3d, lower panel). Taken together, these results clearly indicate that treatment with selumetinib had a significant impact on reducing mesenchymal features such as cell migration and EMT marker expression in both OR cells, but impacted ITGB1 levels in a completely opposite direction. This different response on ITGB1 probably indicate that additional factors are involved in the modulation of ITGB1 in H1975/OR cells thus suggesting that this integrin may be involved in alternative pathways of resistance.

### Role of DDRs and GOF p53 in mediating cell survival and ITGB1 activation

**Figure 4:**
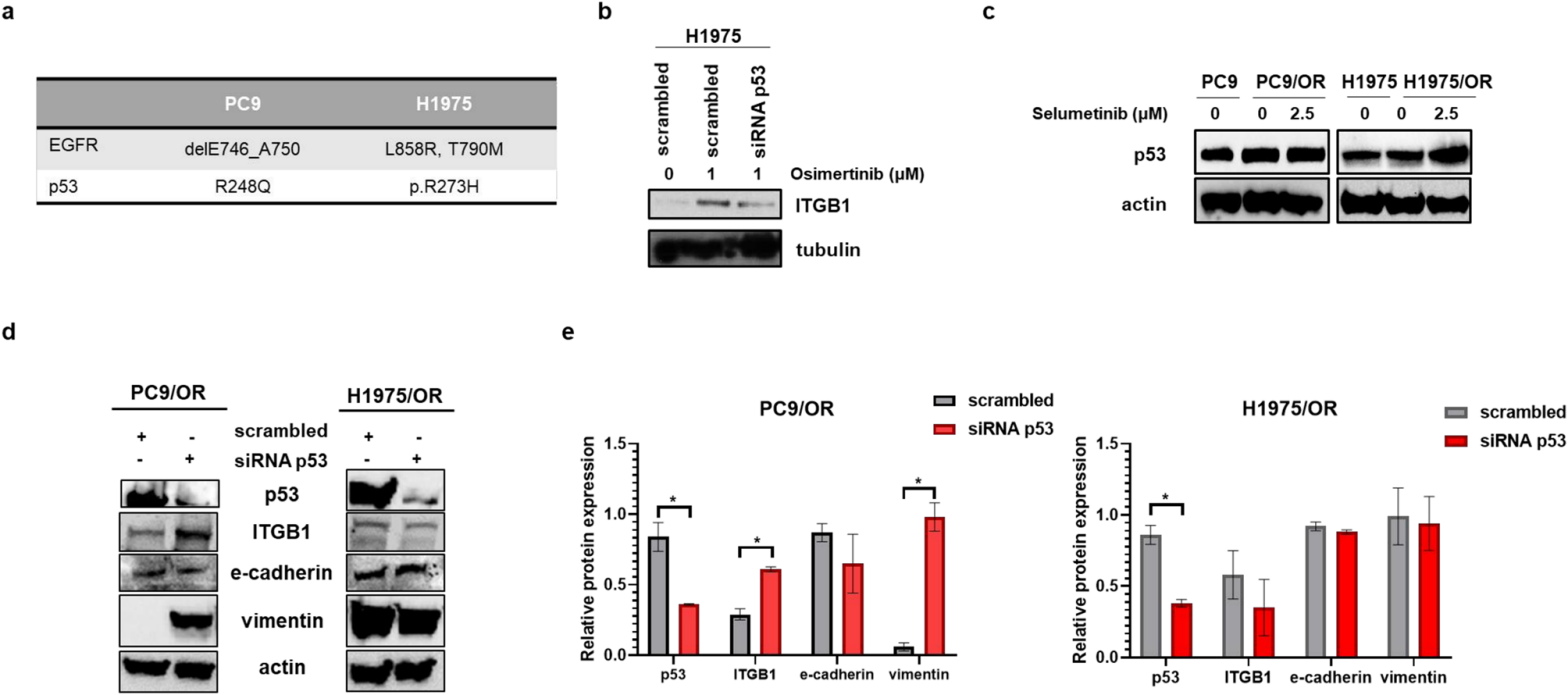
Effect of p53 mutational status in NSCLC cells. (**a**) Characteristics of the NSCLC cell lines used in this study with EGFR and p53 mutational status. (**b**) Levels of ITGB1 in H1975 cells were transfected with siRNA scrambled and siRNA p53 treated or not with osimertinib. (**c**) Western blot analysis of p53 expression in untreated and treated PC9, PC9/OR, H1975 and H1975/OR cells for 72 h. (**d**) Representative western blot images of p53, ITGB1, e-cadherin and vimentin in scrambled and siRNA p53 OR cells. (**e**) Quantitative analysis of gel bands by morphodensitometric analysis using ImageJ software. Data are expressed as relative protein levels of each treated sample compared to the corresponding untreated control. Significant differences versus untreated were indicated with *p < 0.05. At least three independent experiments were performed. Tubulin and actin were used to ensure equal loading.

Considering the strong effect induced by treatment with selumetinib 2.5 µM for 72 h on inhibition of MAPK signaling, EMT and cell migration in both OR cell lines, we wanted to investigate the molecular mechanisms underlying the upregulation of ITGB1 downstream signaling pathway in treated H1975/OR cells and test combinatorial approaches to improve cell toxicity in OR cell lines. In this respect, our previous study reported the relevant role of p53 in regulating the acquisition of EMT signature in H1975 cells after stimulation with Notch1 agonist^35^. Somatic mutations of p53 tumor suppressor gene have been detected in human NSCLC cell lines as reported in Fig. 4a. However, the relationship between p53 mutational status and acquired resistance in NSCLC is inadequately described. Based on these evidences we investigated the role of such transcriptional factor in modulating the expression of ITGB1 in untreated and treated parental cells. In particular, to demonstrate the existence of a cause-effect relationship between ITGB1 levels and p53 expression, we transiently downregulated p53 in H1975 cells, by selective siRNA. Western blotting of whole cell lysates showed an increase of ITGB1 in siRNA scrambled treated with osimertinib for 72 h, whereas in siRNA p53 treated-cells a significant reduction of ITGB1 levels were observed (Fig. 4b). These findings suggested us that p53 was able to promote the integrin upregulation in response to osimertinib treatment in H1975 cells. Moreover, we found that treatment with selumetinib is able to cause an increase of p53 levels in H1975/OR but not in PC9/OR cells (Fig. 4c). These results may be explained considering that mutant p53 in H1975 cells has oncogenic properties, so called gain-of-function (GOF), which favor the transcription of several mediators driving invasion and metastasis also by promoting integrin recycling^36^. To clearly confirm this hypothesis, we downregulated p53 in OR cells and we observed that ITGB1 was significantly upregulated in PC9/OR cells whereas ITGB1 was mild reduced in H1975/OR cells (Fig. 4d, e). Moreover, p53 downregulation induced an increase of EMT signature in PC9/OR cells, while in H1975/OR cells reduced levels of p53 do not affect significantly EMT markers. Thus, these results confirm our hypothesis about different role of p53 in the two models of OR cell lines. In particular, in PC9/OR cells p53 has a role in impairing EMT signature whereas in H1975/OR we suppose that its function in sustaining EMT signaling is mediated by its interaction with other proteins therefore P53 downregulation alone is not enough to obtain EMT reversion. Formation of protein complexes is recognized as a crucial element in carrying out oncogenic functions by mutant P53 that lost their tumor suppressor activity^37^.

**Figure 5:**
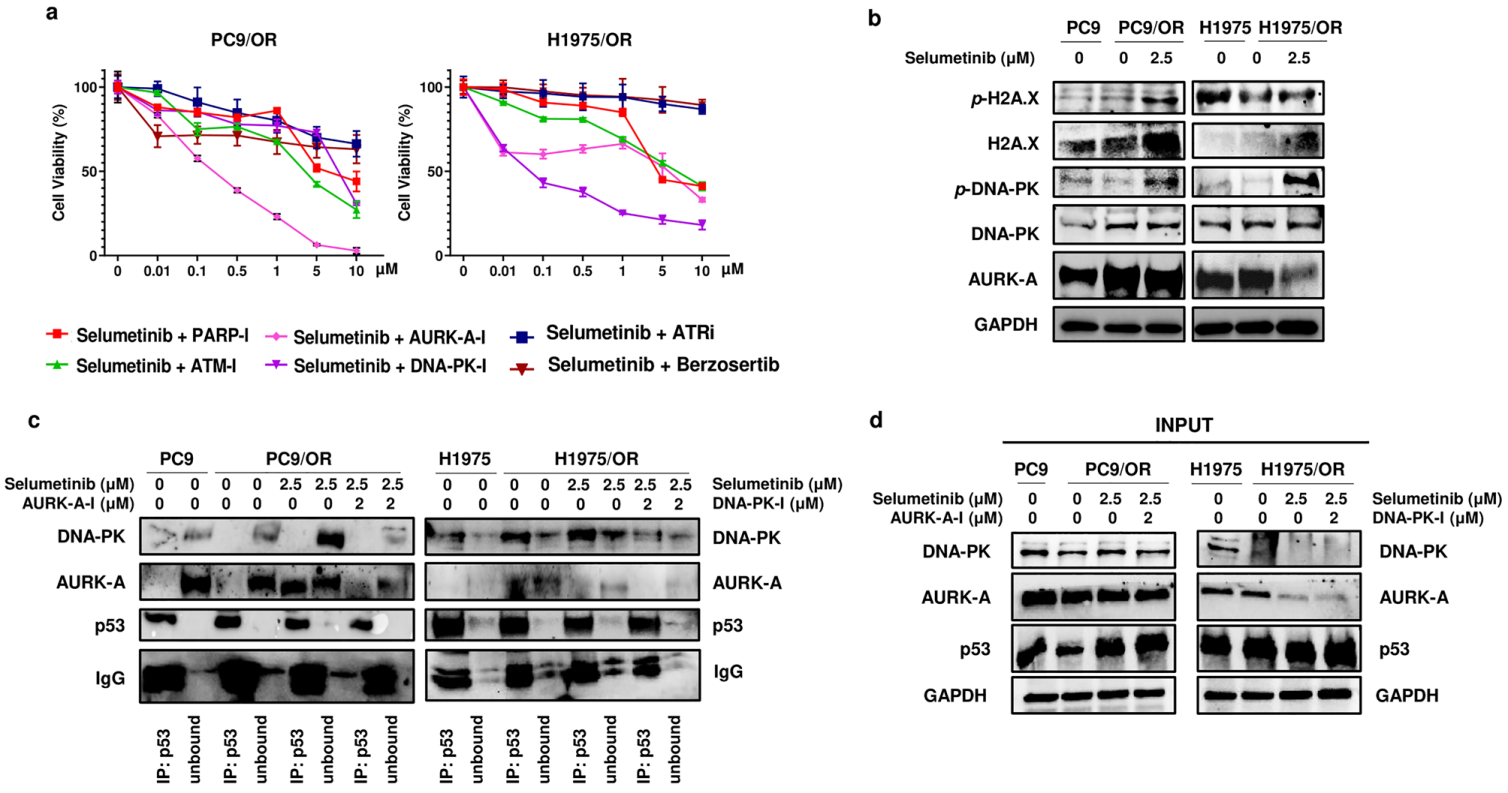
DDR pathway contributes to osimertinib resistance in selumetinib-treated NSCLC cells. (**a**) OR cell toxicity assay in response to increasing concentrations of DDRi (PARP-I, ATM-I, DNA-PK-I, AURK-A-I, ATR-I, benzosertib) in combination with selumetinib (2.5 μM) for 72 h. (**b**) Levels of DDR markers (p-DNA-PK, DNA-PK, p-H2A.X, H2A.X, AURK-A) in response to treatment with selumetinib (2.5 µM) for 72 h. GAPDH was used to ensure equal loading. (**c**) Immunoprecipitation assay with anti-p53 antibody in whole cell lysates and (**d**) representative western bot images of DNA-PK, AURK-A and p53 in input samples from PC9, PC9/OR, H1975 and H1975/OR treated or not with selumetinib (2.5 μM) alone or in combination with AURK-A-I (2 μM) or DNA-PK-I (2 μM), respectively. Tubulin was used to ensure equal loading.

Numerous studies also reported another relevant role of p53 in modulating expression and activity of DNA damage repair (DDR) family proteins^38–40^. In this regard, we hypothesized that mutant p53 might interact with proteins that mediate and enhance DNA damage repair mechanisms as well as maintain ITGB1 overexpression and the activation of its signaling pathway in H1975/OR cells. In this respect, we treated OR cells with PARP (PARP-I), ATM (ATM-I), berzosertib (ATR/ATM-I), ATR (ATR-I), DNA-PK (DNA-PK-I, peposertib) or AURK-A (AURK-A-I) inhibitors at different doses alone or in combination with selumetinib 2.5 µM for 72 h and then we performed MTS assay. Treatment with DDRi alone did not affect cell viability (Supplementary Figure S3 and Table S1). Whereas only the addition of AURK-A-I and DNA-PK-I to selumetinib induced the highest decrease on cell viability in PC9/OR (IC50 = 0.21 µM) and H1975/OR (IC50 = 0.12 µM) cells, respectively (Fig. 5a). These results evidenced that the two OR cell lines differ in sensitivity and specificity to DDRi. Afterwards, we tested DDR protein levels in the two OR cell lines and explored whether these proteins may contribute to cell survival after selumetinib treatment. Figure 5b showed an increase of phosphorylated and total forms of H2A.X and DNA-PK, two well-established markers of DNA damage, in both OR-treated cells compared to untreated ones. In addition, we found that treated PC9/OR cells also exhibited higher AURK-A levels then untreated ones. Moreover, we examined p-ATM/ATM, p-ATR/ATR, PARP and p-Chk2 levels in both OR cell lines in response to selumetinib treatment (Supplementary Figure S4). In H1975/OR but not in PC9/OR we found increased phosphorylated and total ATM. These results are in agreement with previous studies showing that activated DNA damage checkpoint signaling promotes GOF-p53 stabilization through ATM-mediated regulation^41,42^.

Consequently, we performed immunoprecipitation (IP) assay in untreated and selumetinib-treated cells to evaluate protein–protein interaction between p53 and DDR family proteins that provided the highest efficacy in reducing viability when combined with selumetinib (Fig. 5c). We performed such assay by using an anti-p53 antibody and we found an enrichment of the interaction between p53 and DNA-PK in H1975/OR cells as compared to parental cell line. Moreover, we found that p53/DNA-PK complex was not disrupted by selumetinib treatment and that GOF p53 failed to interact with AURK-A in H1975 and H1975/OR. These data clearly demonstrated that GOF p53 can specifically interact with DNA-PK in EGFR-mutant OR NSCLC cells to enhance DNA repair pathways thereby supporting cell survival. Otherwise, no complexes between p53 and DNA-PK could be detected in PC9 and PC9/OR cells. Interestingly, we found a physical interaction between p53 and AURK-A in the PC9/OR cell line after treatment with selumetinib, but not in untreated cells. The input control of the IP experiment samples is shown in Fig. 5d. Taken together, our data demonstrate a selective interaction of p53 with different DDR family proteins, in the two different OR cell lines.

### Effect of combined treatment of DDR inhibitors with selumetinib on cell death

**Figure 6:**
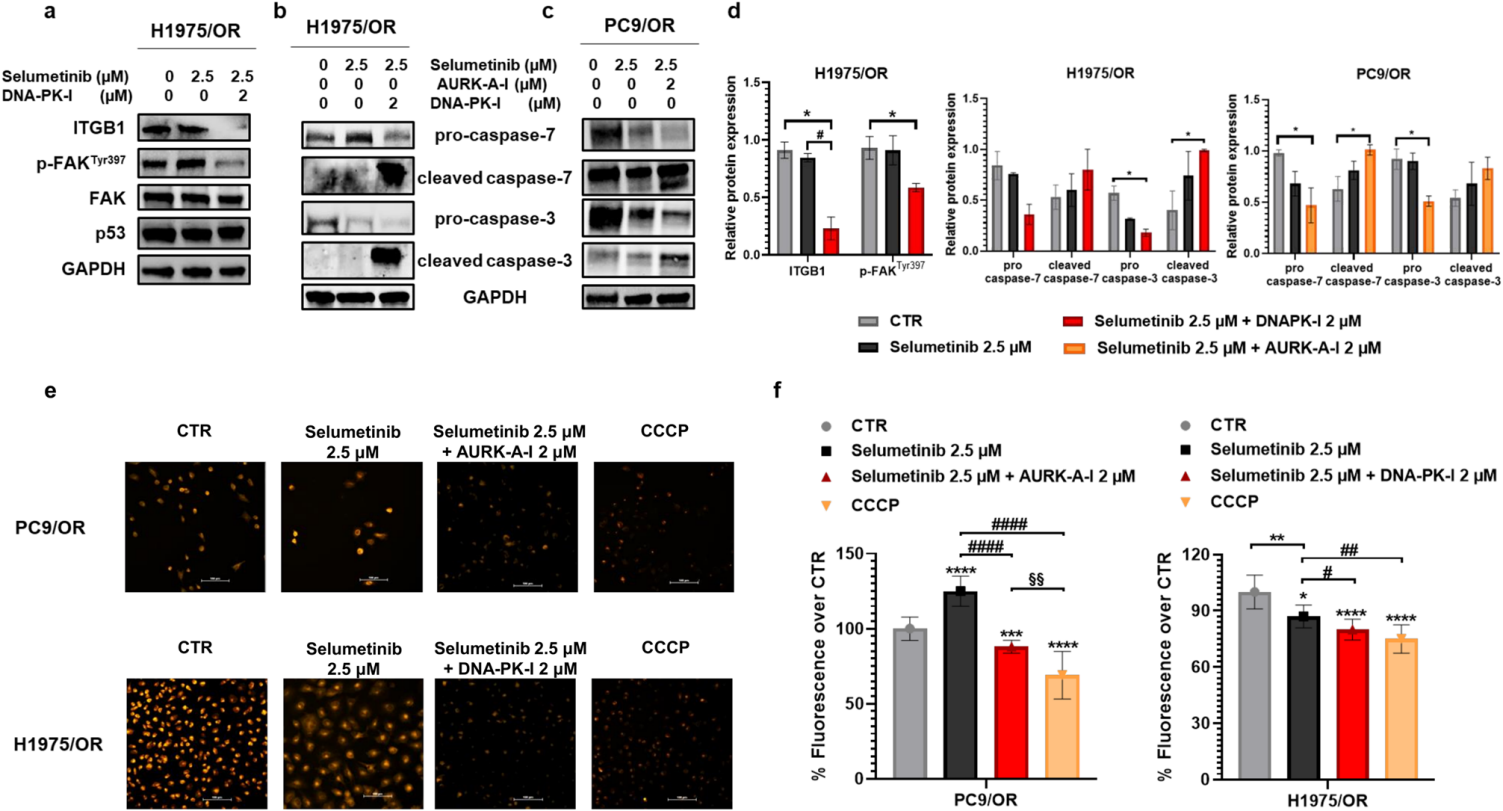
Selective DDR targeting synergizes with selumetinib and strongly affects OR cell viability. (**a**–**c**) Western blotting was performed to measure the expression of (**a**) ITGB1, p-FAK^Tyr397^, FAK and apoptosis-associated proteins caspase-3, cleaved-caspase-3, caspase-7 and cleaved-caspase-7 in (**b**) H1975/OR and (**c**) PC9/OR cells following treatment with selumetinib alone or in combination with DNA-PK-I or AURK-A-I for 72 h, respectively. (**d**) Quantitative analysis of gel bands by morphodensitometric analysis using ImageJ software. Data are expressed as relative protein levels of each treated sample compared to the corresponding untreated control. Significant differences versus untreated were indicated with *p < 0.05. (**e**) Representative live images of PC9/OR and H1975/OR cells stained with TMRE to visualize MMP with a fluorescence microscope. Its depolarization was observed (20× magnification) after cells were exposed to selumetinib (2.5 μM) alone or in combination with DNA-PKI (2 μM) or AURK-A-I (2 μM) for 72 h, or CCCP (as positive control). (**f**) Quantitative analysis of fluorescent intensity was expressed as % relative to untreated cells and expressed as mean ± SE. Statistical significance *p < 0.05, ***p < 0.001 and ****p < 0.0001 versus CTR; ^#^*p* < 0.05, ^##^*p* < 0.01, ^####^*p* < 0.0001 versus selumetinib; ^§§^*p* < 0.01 versus CCCP. At least three independent experiments were performed.

Interestingly, combination treatment of selumetinib plus DNA-PK-I was able to cause a significant reduction of ITGB1 and its downstream signaling pathway (p-FAK^Tyr397^) in H1975/OR cells (Fig. 6a). This relevant result indicates the existence of the interplay, DNA-PK-dependent, between DDR and ITGB1 pathways in GOF p53 cells. We also tested whether the combination of selumetinib with selected DDRi could improve cell death compared to selumetinib alone. According to MTS findings, we investigated the apoptotic machinery in response to combined treatments. In particular, the reduction of pro-caspase 3 and 7 and the significant increase of their respective cleaved forms suggested the activation of apoptotic pathways in H1975/OR (Fig. 6b) and PC9/OR (Fig. 6c). Figure 6 shows quantitative analysis of gel bands by morphodensitometric quantification. To better confirm the apoptosis activation, mitochondria membrane potential (MMP) level was assessed by TMRE staining. A strong reduction of fluorescence intensity of OR cells treated with the combination of selumetinib plus DDRi, indicating MMP decrease, was observed in live fluorescence microscope imaging analysis (Fig. 6e). Quantitative analysis of TMRE fluorescence (%) in PC9/OR cells treated with selumetinib alone or in combination with AURK-A-I showed a reduction in MMP from 125 to 88%. Similarly, the addition of DNA-PK-I to selumetinib reduced TMRE fluorescence (%) from 100 to 80% in comparison to the untreated control (Fig. 6f). Taken together, these results indicate that selumetinib in combination with AURK-A-I or DNA-PK-I can restore sensitivity in OR cells and inhibit tumour growth and survival.

## Discussion

Recent studies support the emergence of EMT and its association with an impaired DDR pathway among the broad mechanisms linked to osimertinib resistance, the new standard of care for all EGFR-mutant NSCLC patients^25,43^. EMT involves upregulation of ITGB1 and activation of downstream pathways (NF-kB, PI3K, Src, Ras-MAPK) to facilitate migration and metastasis. ITGB1 has also been implicated in enhanced chemo- and radio-resistance through modulation of DDR. Defining the ITGB1/DDR cooperation to OR may provide a basis for their use as diagnostic biomarkers or therapeutic targets in EGFR-mutant NSCLC. However, the crosstalk between EMT and DDR pathways is unknown. In this respect, we report that ITGB1-DDR interplay contributes to OR NSCLC tumour progression. In particular, we found that PC9/OR and H1975/OR cells displayed an aggressive phenotype with increase in migration ability and EMT markers (SNAIL, MMP9, TACE, vimentin) compared to parental cells. In addition, we observed that the ability to repair DNA damage was impaired in OR cells, demonstrated by increased levels of γH2A.X. The oncogene-driver EGFR was strongly downregulated, whereas the MAPK pathway (p-ERK1/2, p-MEK1/2, p-p38) remained fully active in PC9/OR and H1975/OR. The activation of MAPK pathway is known to be a biomarker of resistance to EGFR blockade and we have previously demonstrated that combination of osimertinib with selumetinib may delay or overcome resistance to EGFR inhibition with osimertinib alone^44^. Combination of such two inhibitors has also been investigated in phase II clinical trial (NCT03392246) in EGFR mutant NSCLC cancer, and preliminary signal of activity were seen in phase I trial in a subgroup of patient, negative for MET expression^45^. These clinical results underline the relevant role of heterogeneous mechanisms in EGFR resistance and suggest the importance to investigate mechanisms related or alternative to MAPK signaling. Among EMT pathways, high levels of ITGB1, confirmed by both flow cytometry and western blotting, together with activation of its intracellular signaling (p-FAK, p-STAT3), contributed to persistence/reactivation of MAPK pathway. MAPK inhibition with selumetinib reduced migration, EMT markers and p38 levels in both OR cell lines. ITGB1 total expression by western blot was significantly reduced in selumetinib-treated PC9/OR, but not in H1975/OR cells. It is interesting to note that high expression of ITGB1 along with its overactivation has been found in OR cells even if in a context of potential sub-clonal heterogeneity under treatment pressure, thus concurring along with MAPK and DDR activation to osimertinib-resistance. We speculate that high β1 levels along with its signaling overactivation may be considered as a marker of osimertinib resistance. Moreover, β1 may heterodimerize with different α subunits in promoting cancer progression and the role of each different integrin heterodimers will be object of future studies to better dissect this mechanism.

**Figure 7:**
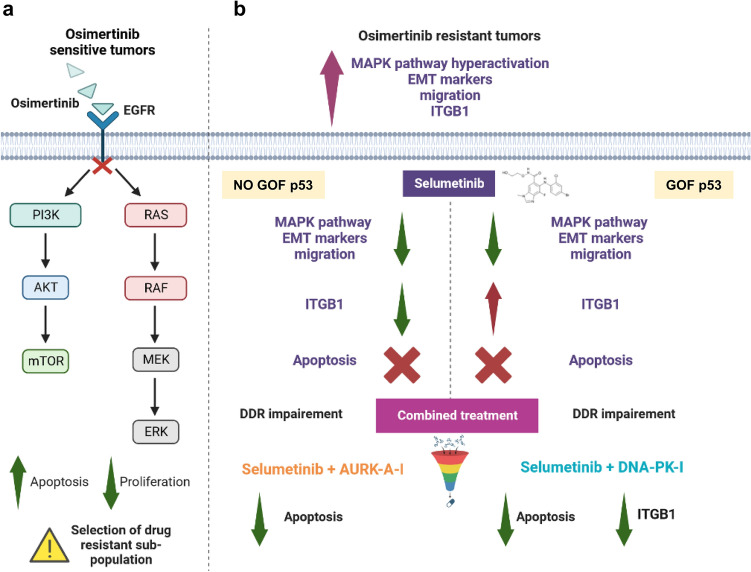
A proposed graphical summary of new personalized therapy treatments. (**a**) Osimertinib tumors responded to the efficient TKI but a selection of drug resistant sub-population appears. (**b**) Osimertinib resistant tumors showed more aggressive phenotype and different personalized therapeutic approach will be necessary based on the mutational status of p53. The graphical summary was produced by the authors using free BioRender platform.

Our findings indicate that p53 mutations can influence the acquired resistance to osimertinib, through the sustenance of genomic instability, that may require the targeting of DDR pathways. Infact, tumor suppressor p53 that is known as a “guardian of the genome” and functions as an important protein in inhibiting cancer cell growth is also activated in response to DNA damage triggering to the induction of apoptosis and subsequently leading to cancer inhibition^39^. However it is well known that, the presence of p53 mutations can contribute to progression of a cancer cell by both loss of tumor suppressor activity and by acquisition of oncogenic properties favoring changes in tumor phenotype and promoting cancer invasion^46^. In this respect, it has also been reported a direct contribution of p53 in sensitivity to EGFR TKIs in NSCLC and that changes in p53 status affected primary sensitivity as well as acquired resistance to EGFR-TKIs according to cell type. In particular, p53 silencing did not affect primary and acquired resistance to EGFR-TKIs in PC9 cells, but it led to enhance sensitivity to osimertinib through the EMT impairment in H1975 cells^40^. Here, we report a novel p53 status-dependent molecular and functional link of ITGB1 with EGFR-TKI resistance. We show that loss of function of p53 empowers ITGB1 signaling during EMT. Selumetinib affects ITGB1 signaling and migration ability in a p53-dependent manner in NSCLC-OR cell lines. Moreover, our results show that novel crosstalk exist between ITGB1 and DDR in NSCLC and play an important role in the inhibition of both cancer migration and cell proliferation. Functionally, combination of selumetinib plus p53 status-dependent DDRi suppress cell viability in OR NSCLC cell lines. Taken together, our results describe novel molecular and functional insights into the anti-EMT effect of selumetinib via an ITGB1-dependent pathway in some OR NSCLCs. ITGB1 may be a new therapeutic target to be explored along with p53 status in NSCLC, which may improve treatment and prognosis of NSCLC patients. As example, the efficacy of combination of selumetinib plus DNA-PK-I in vitro in overcoming resistance to osimertinib may be explored in clinical context for patients harbouring mutations of p53 (Fig. 7).

In conclusion, our data suggest that high levels of ITGB1 and its overactivation may represent a biomarker of tumor invasion and progression after prolonged therapy with osimertinib and also with inhibitors of MAPK pathway like selumetinib. In particular, the susceptibility of OR cells to DNA damage repair may provide potential options for using appropriate DDR inhibitors in combination with selumetinib in OR patients with different mutational status of p53. Moreover, since DDR modulation is highly correlated with immune-responsiveness features in NSCLC, future studies may investigate novel immunotherapy combinations.

## Methods

### Reagents

Antibodies for western blotting were purchased from Cell Signaling (Danvers, MA): p-EGFR (Tyr1068) (2234, 1:1000), EGFR (4267, 1:1000), p-MEK1/2 (Ser217/221) (9154, 1:1000), MEK1/2 (8727, 1:1000), p-p44/42 MAPK (Erk1/2) (Thr202/Tyr204) (9101, 1:1000), p44/42 MAPK (Erk1/2) (9102, 1:1000), p-p38 MAPK (Thr180/Tyr182) (9211, 1:1000), p38 MAPK (9212, 1:1000), α-tubulin (2144, 1:2000), vimentin (3390, 1:1000), TACE (3976, 1:1000), Snail (3879, 1:1000), E-cadherin (3195, 1:1000), p-FAK (Tyr397) (3283, 1:1000), FAK (3285, 1:1000), p-STAT3 (Tyr705) (9131, 1:1000), STAT3 (9139, 1:1000), GAPDH (2118, 1:2000), β-actin (3700, 1:2000), p-DNA-PK (68716, 1:1000), DNA-PK (38168, 1:1000), PARP (9532, 1:1000), p-Histone H2A.X (Ser139) (9718, 1:1000), Histone H2A.X (2595, 1:1000), p-Chk2 (Thr68) (2197, 1:1000), Chk2 (2662, 1:1000), p53 (2524, 1:1000), caspase-7 (9492, 1:1000), caspase-3 (14220, 1:1000), AURK-A (14475, 1:1000); Santa Cruz Biotechnology (Dallas, TX): integrin β1/ITGB1 (sc-374429, 1:1000). Selumetinib (AZD6244, MEK1/2 inhibitor), osimertinib (AZD9291, EGFR^T790M^), M3814 (DNA-PK inhibitor, DNA-PK-I) and ZM-447439 (AURK-A inhibitor, AURK-A-I), MK-4827 (PARP inhibitor, PARP-I), M4076 (ATM inhibitor, ATM-I), M6620 (ATM/ATR inhibitor, benzosertib) and M4344 (ATR inhibitor, ATR-I) were purchased from Selleck Chemicals (Selleckchem).

### Cell lines and treatments

Two NSCLC H1975 and PC9 cell lines were obtained from and authenticated by the American Type Culture Collection (ATCC, Manassas, VA, USA). PC9 and H1975 cells were continuously exposed to osimertinib to establish the corresponding two osimertinib-resistant (OR) cell lines as previously described^47^. All cell lines were grown in RPMI 1640 (Gibco) medium supplemented with 10% fetal bovine serum (FBS), 100 IU/mL penicillin and 50 µg/mL streptomycin in a humidified incubator in 5% CO_2_ at 37 °C. OR cells are always maintained with osimertinib at a dose lower than their IC50 except during specific experiments. Cells were treated with osimertinib, selumetinib, and DNA-PK-I, AURK-A-I, PARP-I, ATM-I, berzosertib or ATR-I for 72 h at the indicated concentrations.

### MTS assay

Drug-induced toxicity was assessed by using the MTS assay (Cell Counting Kit-8). Briefly, cells were seeded in 96-well flat-bottomed plates at a density of 1,000 cells/well and treated for 72 h with increasing concentrations (0.01–10 µM) of osimertinib, selumetinib, DNA-PK-I, AURK-A-I, PARP-I, ATM-I, berzosertib or ATR-I. The number of viable cells was determined spectrophotometrically and expressed as the percentage of viable cells, considering the untreated control cells as 100%. Concentration that inhibits 50% of cell growth (IC50) was determined by using AAT Bioquest online tools (https://www.aatbio.com/tools/ic50-calculator). At least three independent experiments were performed in triplicates and data were pooled.

### Western Blot

Whole cell lysates were prepared as previously described^48^. Briefly, cells were treated with osimertinib, selumetinib, DNA-PK-I or AURK-A-I alone or in combination at the indicated concentrations for 72 h and then lysed with cold RIPA buffer (0.1% sodium SDS, 0,5% deoxycholate, 1% Nonidet, 100 mM NaCl, 10 mM Tris–HCl (pH 7.4), 0.5 mM DTT, and 0.5% PMSF) added with protease (Hoffmann-La Roche) and phosphatase (PhosSTOP; Roche Diagnostics) inhibitors, incubated 20 min on ice and clarified by centrifugation at 15,000 rpm for 20 min at 4 °C. Then, western blot analysis of proteins from different lysates was performed. Briefly, protein samples were resolved SDS-PAGE gel electrophoresis and transferred onto 0.2 µm nitrocellulose membranes (Trans-Blot Turbo; BioRad). After blocking membranes with selected antibodies, proteins were detected with Clarity Western ECL Substrate using the ChemiDoc (BioRad). Images were analyzed using BioRad software Image Lab 3.0.1.

### Nuclear extracts

For nuclear protein extraction^49^, cells were washed twice in ice-cold PBS and the pellets were suspended in 1 mL of buffer A (10 mmol/L HEPES pH 7.9, 1.5 mmol/L MgCl2, 10 mmol/L KCl, 0.01% Triton X-100, 0.5 mmol/L DTT, and 0.5 mmol/L PMSF). After 20 min on ice, samples were vortexed and nuclei were recovered by centrifugation for 5 min at 5000 rpm at 4 °C, followed by resuspension in 25 μL of buffer B (10 mmol/L HEPES pH 7.9, 1.5 mmol/L MgCl2, 400 mmol/L KCl, 0.2 mmol/L EDTA, 25% glycerol, 0.5 mmol/L DTT, protease inhibitors, and 0.5 mmol/L PMSF). After an additional 20 min on ice, samples were subjected to two cycles of freeze–thaw in liquid nitrogen and then maintained at 4 °C for 5 min. Nuclear extracts were centrifuged for 10 min at 13,000 rpm at 4 °C, and the supernatant was recovered. Western blotting of proteins from both whole cell lysates and nuclear extracts was carried out using a standard procedure. PVDF membranes were probed by using mouse monoclonal antibodies recognizing TWIST (Cell Signaling) and rabbit polyclonal antibodies specific for histone H3 (Abcam). A commercially available ECL kit (Advansta, San Jose, CA, USA) was used to reveal the reaction.

### Immunoprecipitation

For immunoprecipitation assays (IP)^50^, proteins from whole cell lysates (250–500 µg) were incubated overnight at 4 °C with anti-p53 mouse monoclonal antibody (Cell Signaling). The immunoprecipitated proteins recovered by absorption to EZview Red Protein A Affinity Gel (Sigma-Aldrich) were separated by SDS-PAGE, transferred to nitrocellulose membrane and probed for the indicated proteins. Three independent experiments were performed.

### Migration assay

Cells were seeded in 6-well cell culture plates at a density of 150,000 cells/well and allowed to grow. After 72 h, cells were resuspended in 100 μl RPMI supplemented with 1% FBS and seeded on top of the filter membrane with pore size of 8.0 μm in a Transwell insert (Corning Life Sciences, Tewksbury, MA). Cell migration was stimulated by supplementing cell culture medium with 5% FBS, as chemoattractant, in the Transwell lower compartment. After 6 h of migration, cells were fixed with 70% ethanol for 10 min and stained with 0.2% crystal violet solution by incubation at room temperature for further 10 min. Cells on the top of the membrane were gently removed with a cotton tipped applicator and cell numbers moved through the membrane pores were determined by using an inverted microscope equipped with a 10X. Five fields for each membrane were photographed and the number of invading cells were determined. To obtain a quantitative analysis, crystal violet was dissolved in a dH_2_O with 33% acetic acid and then measured spectrophotometrically at 590 nm. Three independent experiments were performed and pooled together.

### RNA interference

TP53-targeted siRNA pool (ON-TARGETplus SMARTpool siRNA TP53) and control non-targeting siRNA pool (CTR) were purchased from Dharmacon Inc. (Lafayette, CO, USA) and used according to the manufacturer’s instructions. Briefly, cell suspension from parental or OR cells were plated at 40% confluence and allowed to attach. Cells were then transfected with 100 nM siRNAs using Dharmafect reagent (Dharmacon). After 24 h cells were treated with osimertinib 1 μM for 72 h. Finally, cells were recovered and then lysed for western blot analysis. Three independent experiments were performed.

### Flow cytometry

For flow cytometry analysis, at least 500,000 cells were incubated with anti-α5β1 rabbit monoclonal primary antibody (Merck Millipore), anti-β1 mouse monoclonal primary antibody (Merck Millipore) or anti-αv mouse monoclonal primary antibody (Merck Millipore) and a secondary FITC-labeled mouse or rabbit monoclonal antibody (Abcam) at 4 °C for 30 min in the dark. For intracellular staining, cells were prefixed and permeabilized with Fix & Perm cell permeabilization Kit (ThermoFisher Scientific) before adding the antibodies. After washing steps, the labeled cells were analyzed by flow cytometry using a BD FACS Canto II (Becton & Dickinson, Mountain View, CA, USA). Analysis was conducted using BD FACSDiva™ Software (BD Biosciences). Flow analyses were carried out with at least 300,000 cells, and each test was performed in triplicate. For all experiments, we used a negative control of cells processed without primary antibody.

### Mitochondria membrane potential

The mitochondria membrane potential (MMP) is depolarized when cells undergo in apoptosis. Thus, MMP is an essential parameter indicating mitochondrial function, and it can be measured using the TMRE dye. Briefly, H1975/OR and PC9/OR cells were seeded in 24 well plates at density of 50,000 cells/well and treated with selumetinib (2.5 μM) plus DNA-PK-I (2 μM) or AURK-A-I (2 μM) for 72 h as described above. Firstly, for the positive control (mitochondria membrane potential loss), CCCP (50 μM) was added to the control wells and incubated for 5 min at 37 °C and 5% CO_2_. Then, all wells were incubated with TMRE staining solution (200 nM) for 20 min in an incubator. Cells were washed with PBS and red fluorescence intensity was measured at Ex/Em: 550/580 nm with a florescence microplate reader (VICTOR® Nivo™ multimode plate reader). In addition, representative images were captured using a fluorescence microscopy equipped with a 20X lens (ECLIPSE Ti2, Nikon, Japan). Three independent experiments were performed.

### Statistical analysis and graphical elaboration

Statistical analyses were performed using the Prism 8 (GraphPad Software, San Diego, CA, USA) software and unpaired Student t test was used as appropriate. A *p*-value < 0.05 was considered to indicate statistical significance. The western blotting signals were quantified by morphodensitometric analysis using ImageJ software (NIH, Bethesda, MD, USA). Briefly, the product of the area and optical density of each band were determined and normalized to the same parameter derived from the equal loading used. Data were expressed as relative protein levels of each treated sample compared to the corresponding vehicle-treated internal control. We have drawn by ourselves the final graphical summary using free BioRender platform.

### Supplementary Information

Supplementary Information.

## Data Availability

The datasets used and analyzed during the current study are available from the corresponding author on reasonable request.
