# Supplementary material for: ITGB1 and DDR activation as novel mediators in acquired resistance to osimertinib and MEK inhibitors in EGFR-mutant NSCLC
Source: Sci Rep. 2024 Jan 4;14:500. doi: 10.1038/s41598-023-50568-5 (PMC10766645; doi:10.1038/s41598-023-50568-5)

**Supplementary Materials for**

**ITGB1 and DDR activation as novel mediators in acquired resistance to osimertinib and MEK inhibitors in EGFR-mutant NSCLC**

Caterina De Rosa^1^, Viviana De Rosa^2^, Concetta Tuccillo^1^, Virginia Tirino^3^, Luisa Amato^1^, Federica Papaccio ^4^, Davide Ciardiello ^5^, Stefania Napolitano ^1^, Giulia Martini ^1^, Fortunato Ciardiello^1^, Floriana Morgillo^1^, Francesca Iommelli^2,†^, Carminia Maria Della Corte^1,†,^

* Correspondence: [carminiamaria.dellacorte@unicampania.it](mailto:carminiamaria.dellacorte@unicampania.it) ; Tel.: +393929160541

**The PDF file includes:**

**Figure S1. FACS analysis of α5β1 and αv in all cell lines**

**Figure S2. MTS of parental cells with selumetinib**

**Figure S3. PC9/OR and H1975/OR MTS cytotoxicity assay in response to DDRi**

**Figure S4. Western blot of PARP, p-Chk2, p-ATM/ATM and p-ATR/ATR in parental and resistant PC9 and H1975**

**Table S1. Cytotoxicity IC50 values of DDR inhibitors in OR NSCLC cell lines**

**Figure S5. Original western blot images included in Figure 1b. Cropped WB bands displayed in Figure 1b are highlighted by a yellow box**

**Figure S6. Original western blot images included in Figure 1c. Cropped WB bands displayed in Figure 1c are highlighted by a yellow box**

**Figure S7. Original western blot images included in Figure 1d. Cropped WB bands displayed in Figure 1d are highlighted by a yellow box**

**Figure S8. Original western blot images included in Figure 2c. Cropped WB bands displayed in Figure 2c are highlighted by a yellow box**

**Figure S9. Original western blot images included in Figure 3b. Cropped WB bands displayed in Figure 3b are highlighted by a yellow box**

**Figure S10. Original western blot images included in Figure 3d. Cropped WB bands displayed in Figure 3d are highlighted by a yellow box**

**Figure S11. Original western blot images included in Figure 4b and c. Cropped WB bands displayed in Figure 4a are highlighted by a yellow box**

**Figure S12. Original western blot images included in Figure 4d. Cropped WB bands displayed in Figure 4c are highlighted by a yellow box**

**Figure S13. Original western blot images included in Figure 5b. Cropped WB bands displayed in Figure 4d are highlighted by a yellow box**

**Figure S14. Original western blot images included in Figure 5c. Cropped WB bands displayed in Figure 5a are highlighted by a yellow box**

**Figure S15. Original western blot images included in Figure 5d. Cropped WB bands displayed in Figure 5b are highlighted by a yellow box**

**Figure S16. Original western blot images included in Figure 6a. Cropped WB bands displayed in Figure 5c are highlighted by a yellow box**

**Figure S17. Original western blot images included in Figure 6b. Cropped WB bands displayed in Figure S2 are highlighted by a yellow box**

**Figure S18. Original western blot images included in Figure 6c. Cropped WB bands displayed in Figure S2 are highlighted by a yellow box**

**Figure S19. Original western blot images included in Figure S4. Cropped WB bands displayed in Figure S2 are highlighted by a yellow box**

**
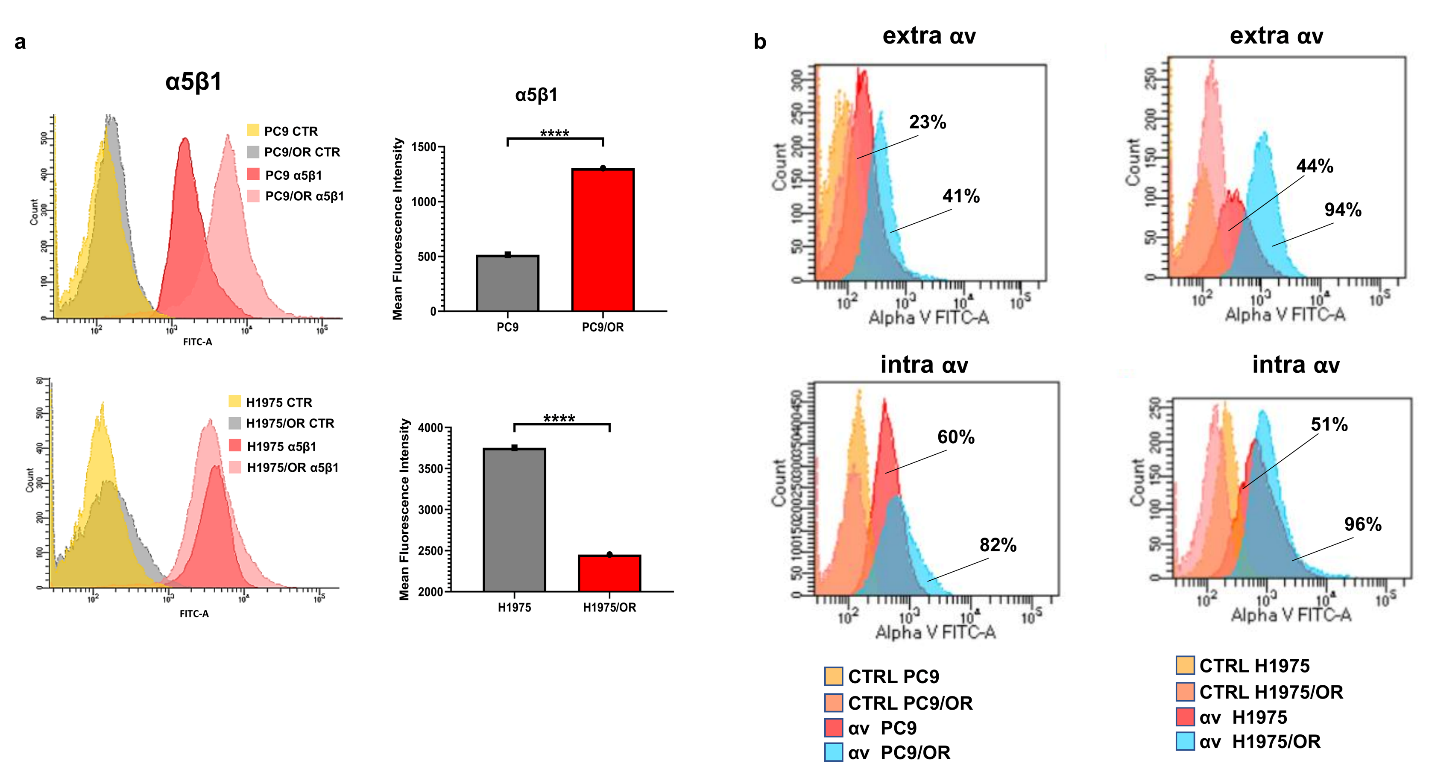
**

**Figure S2 MTS of parental cells with selumetinib.**

**
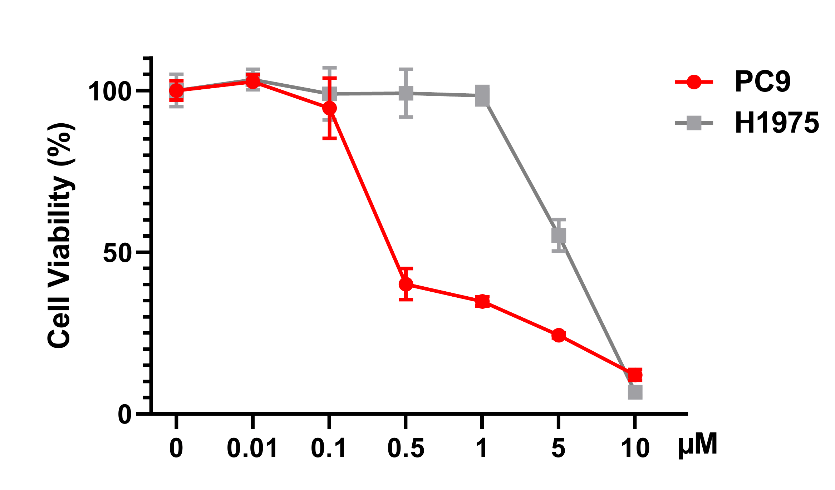
**

**Figure S3.** **PC9/OR and H1975/OR MTS cytotoxicity assay in response to increasing concentrations DDRi (PARP-I, ATM-I, DNA-PK-I, AURK-A-I, berzosetib, ATR-I) for 72 h**

**
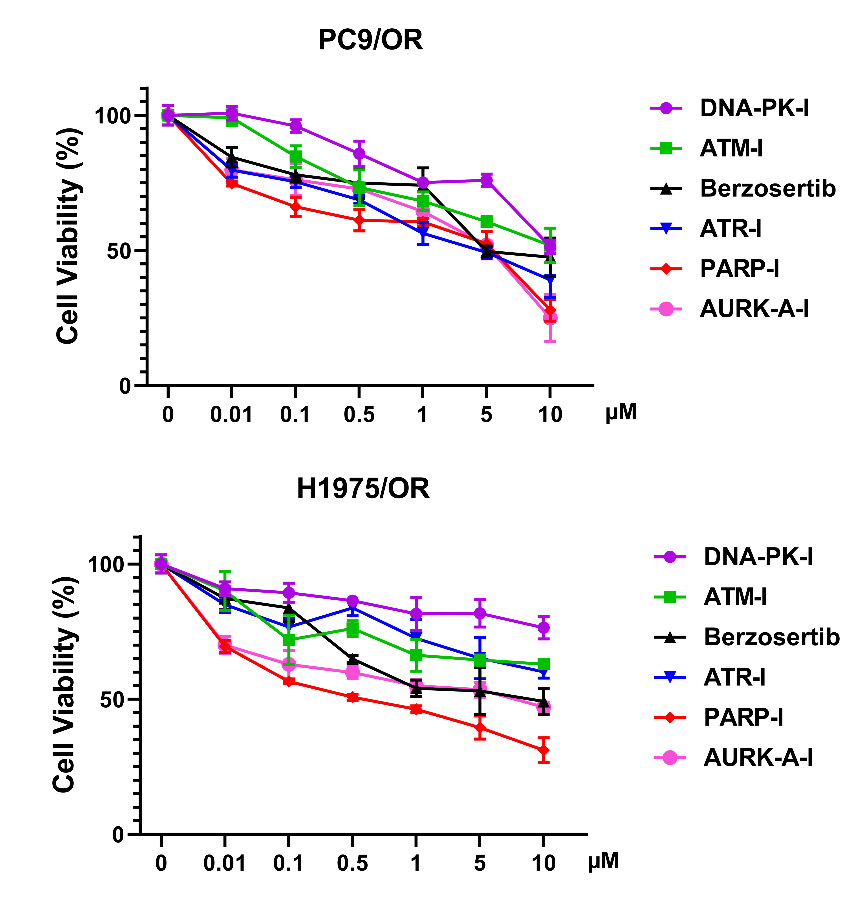
**

**Figure S4. Levels of PARP, p-Chk2, p-ATM/ATM and p-ATR/ATR in response or not to treatment with selumetinib (2.5 µM) for 72 h in parental (PC9, H1975) and resistant (PC9/OR, H1975/OR) cell lines. Tubulin was used to ensure equal loading.**


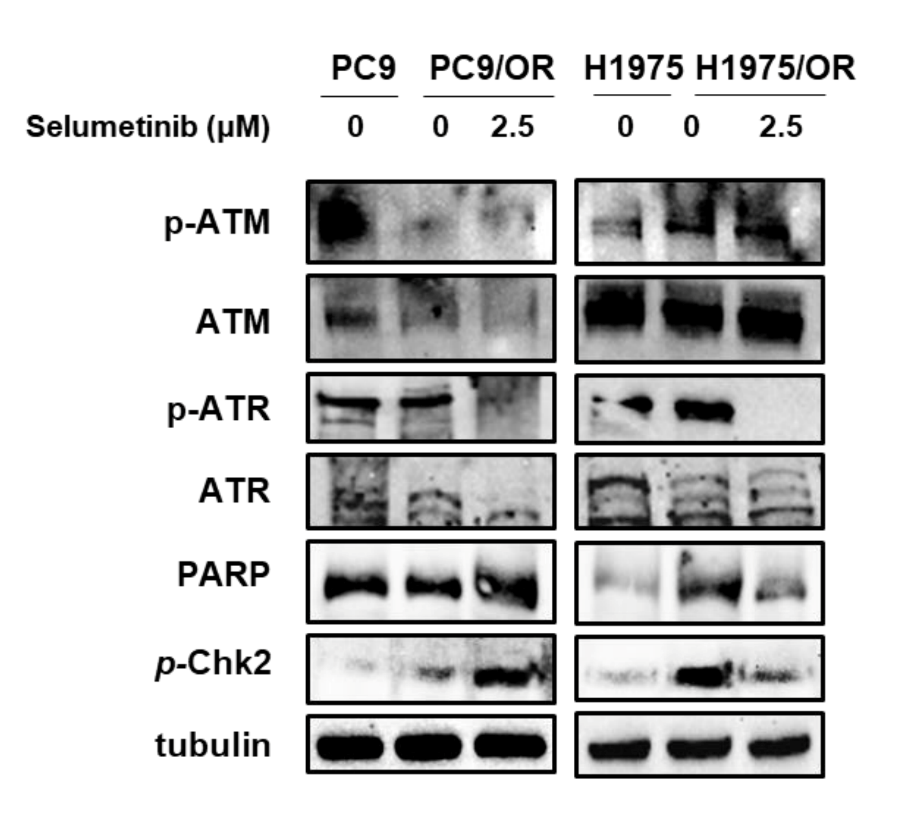


**Table S1.** **Cytotoxicity IC50 values of DDR inhibitors in OR NSCLC cell lines**

| **(µM)** | **PC9/OR** | **H1975/OR** |
| --- | --- | --- |
| **DNA-PK-I** | **10.61** | **24.91** |
| **ATM-I** | **6.735** | **9.522** |
| **Berzosertib** | **5.020** | **3.684** |
| **ATR-I** | **2.723** | **9.914** |
| **PARP-I** | **2.183** | **0.7014** |
| **AURK-A-I** | **2.834** | **2.954** |

**Figure S5.**


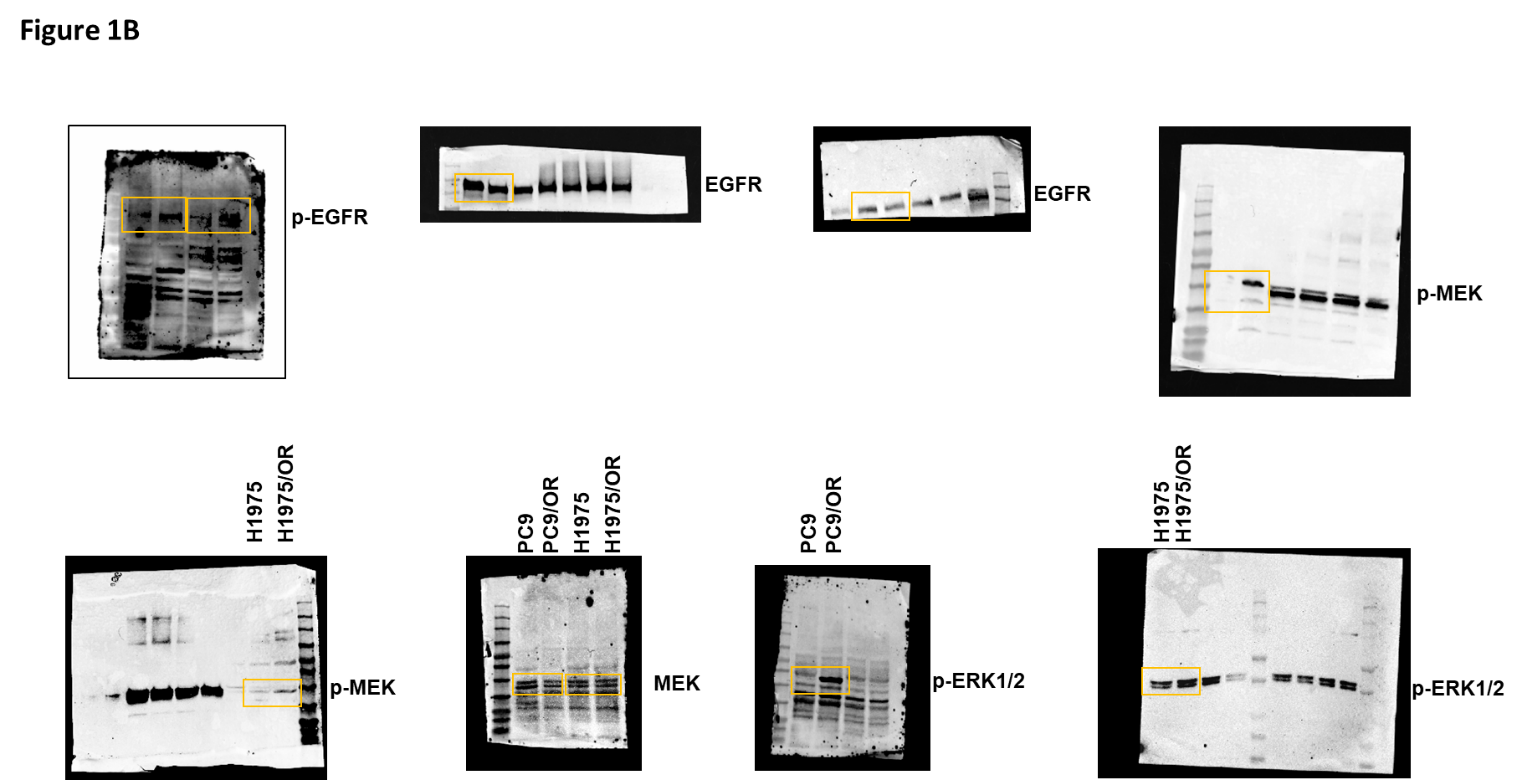


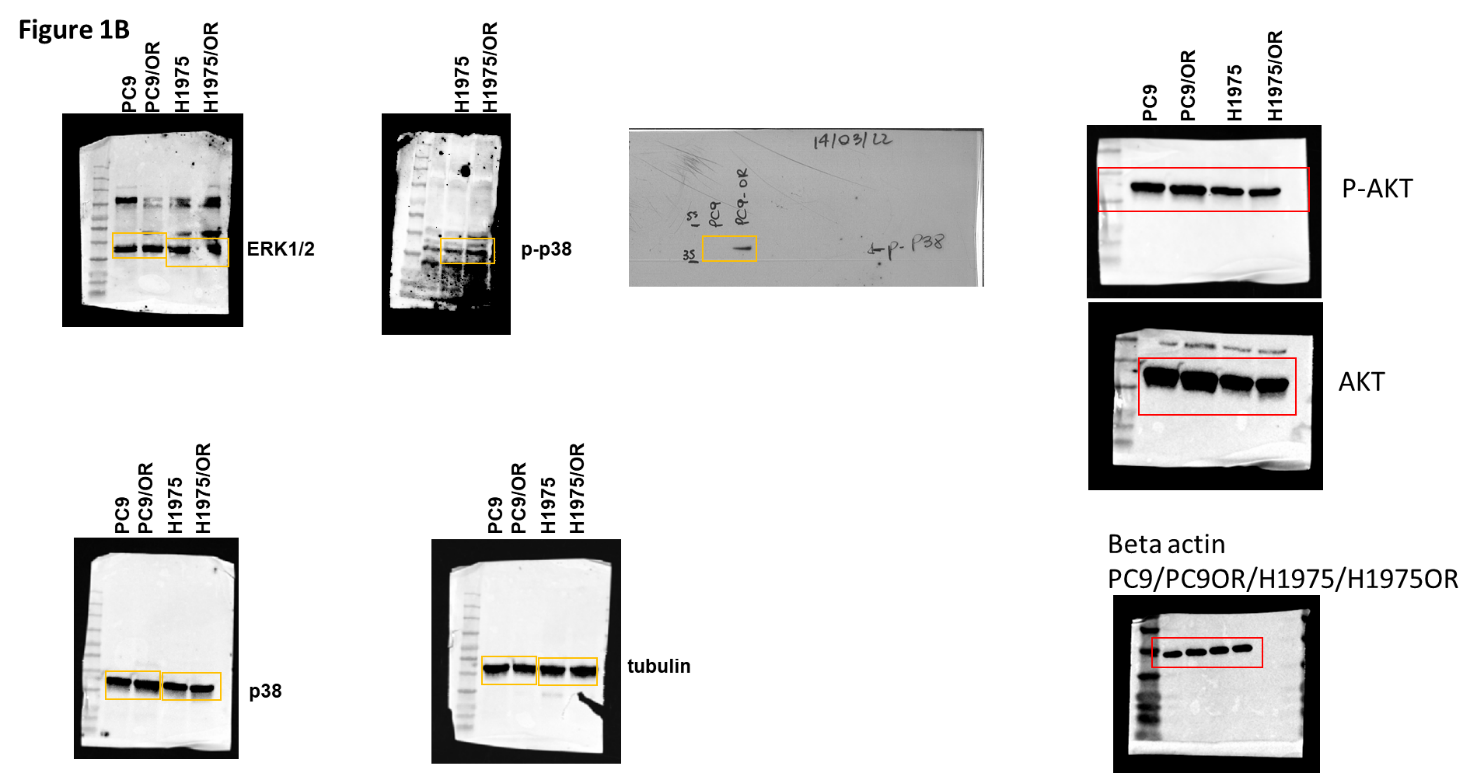


**Figure S6**


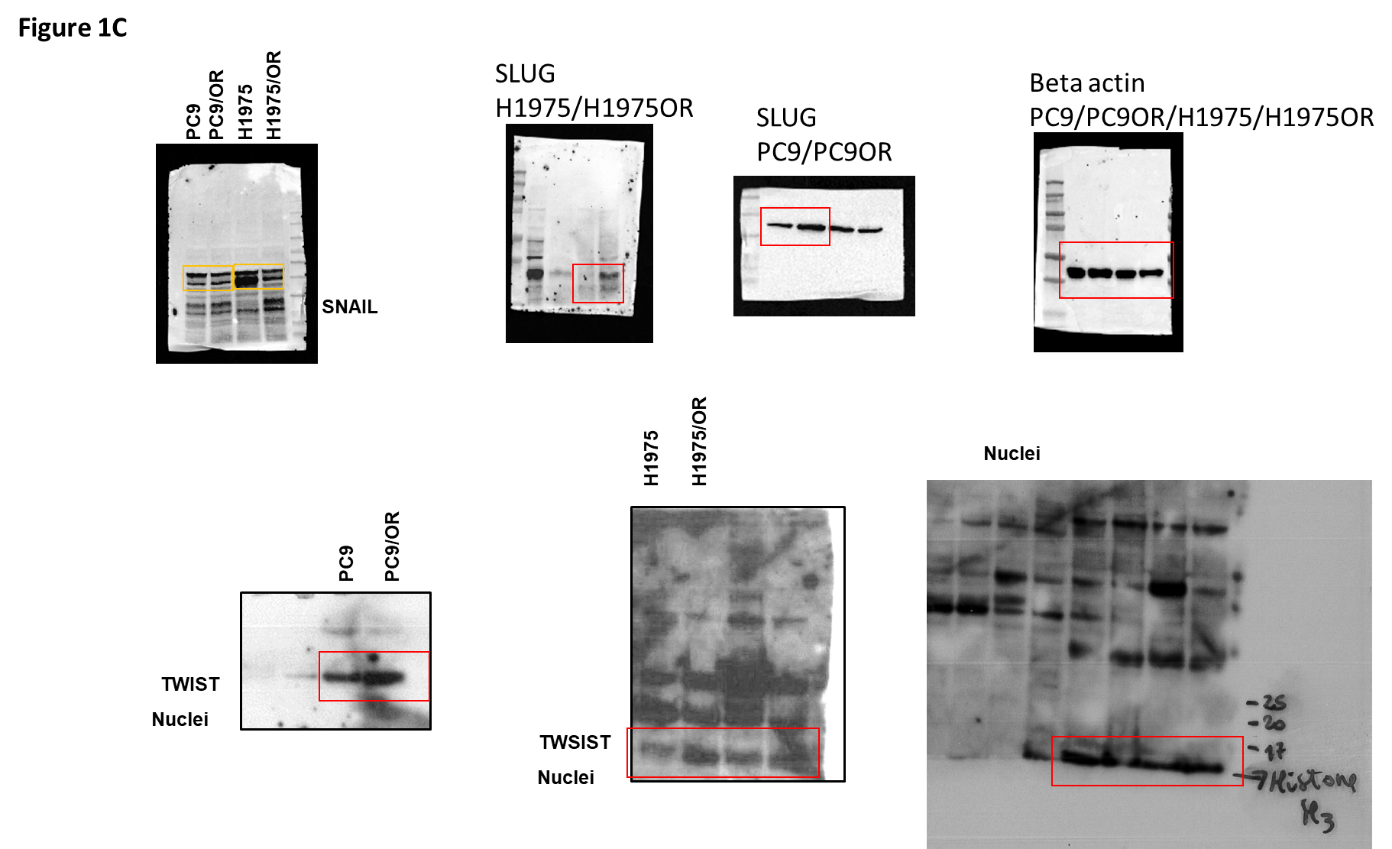


**Figure S7.**


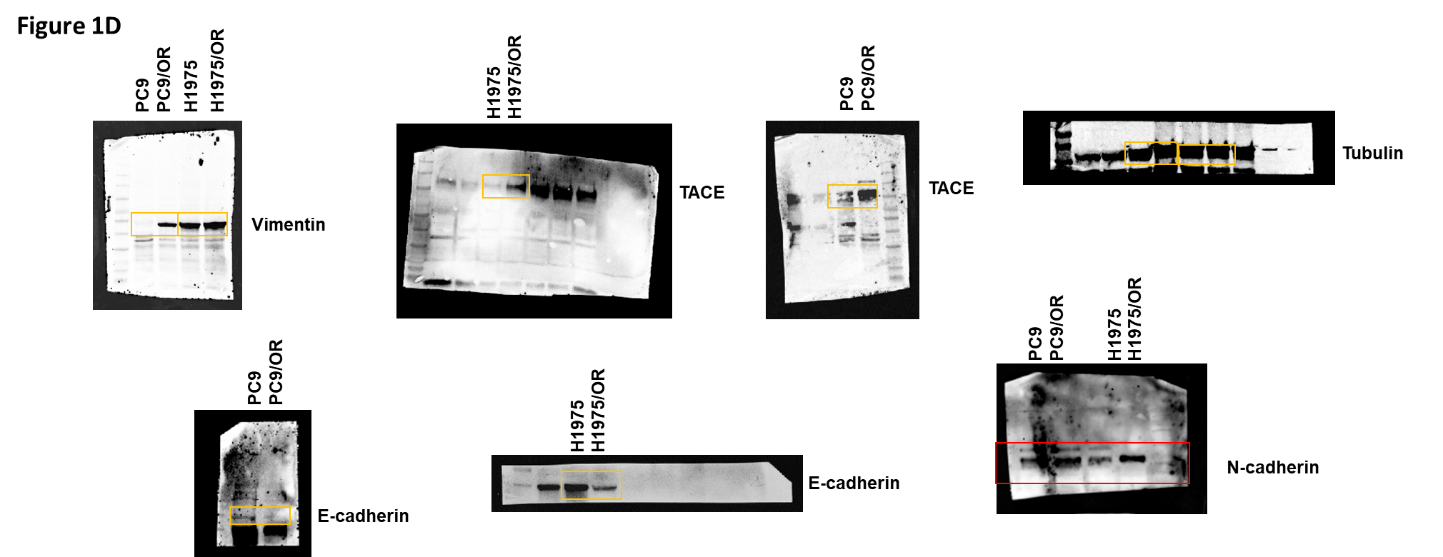


**Figure S8.**


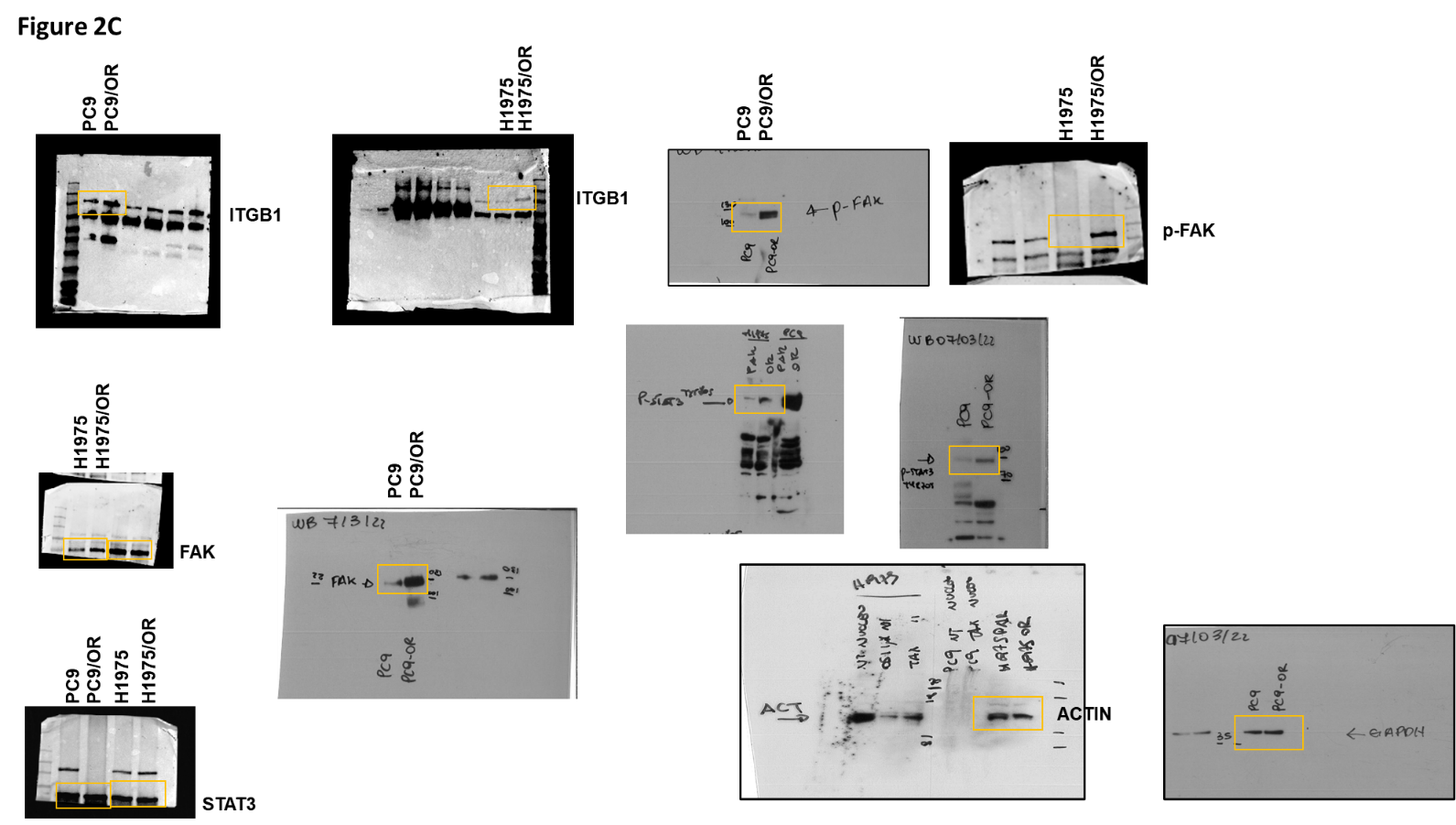


**Figure S9.**

**Figure 3b**
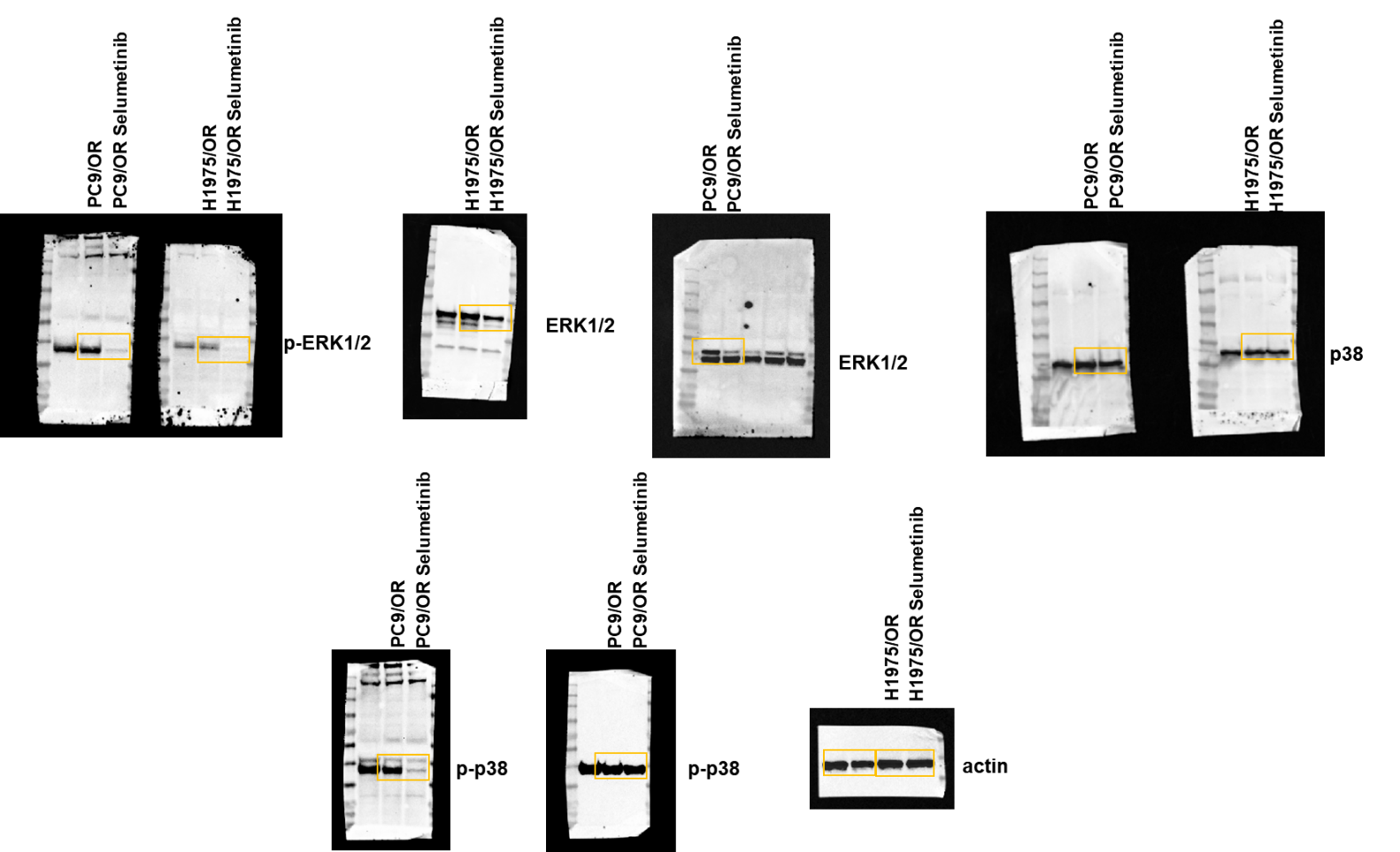


**Figure S10.**


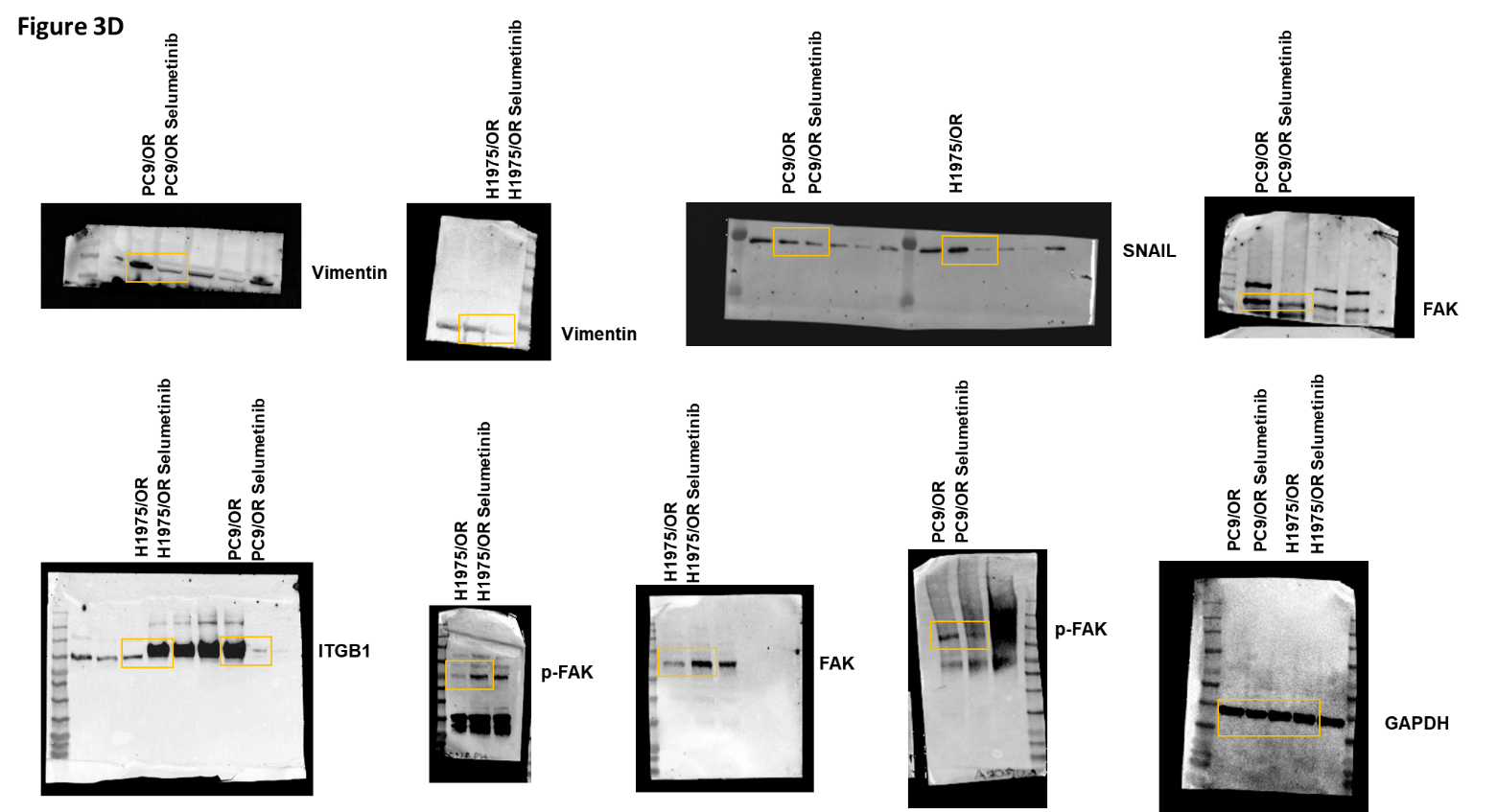


**Figure S11.**

**Figure 4b and c**

**
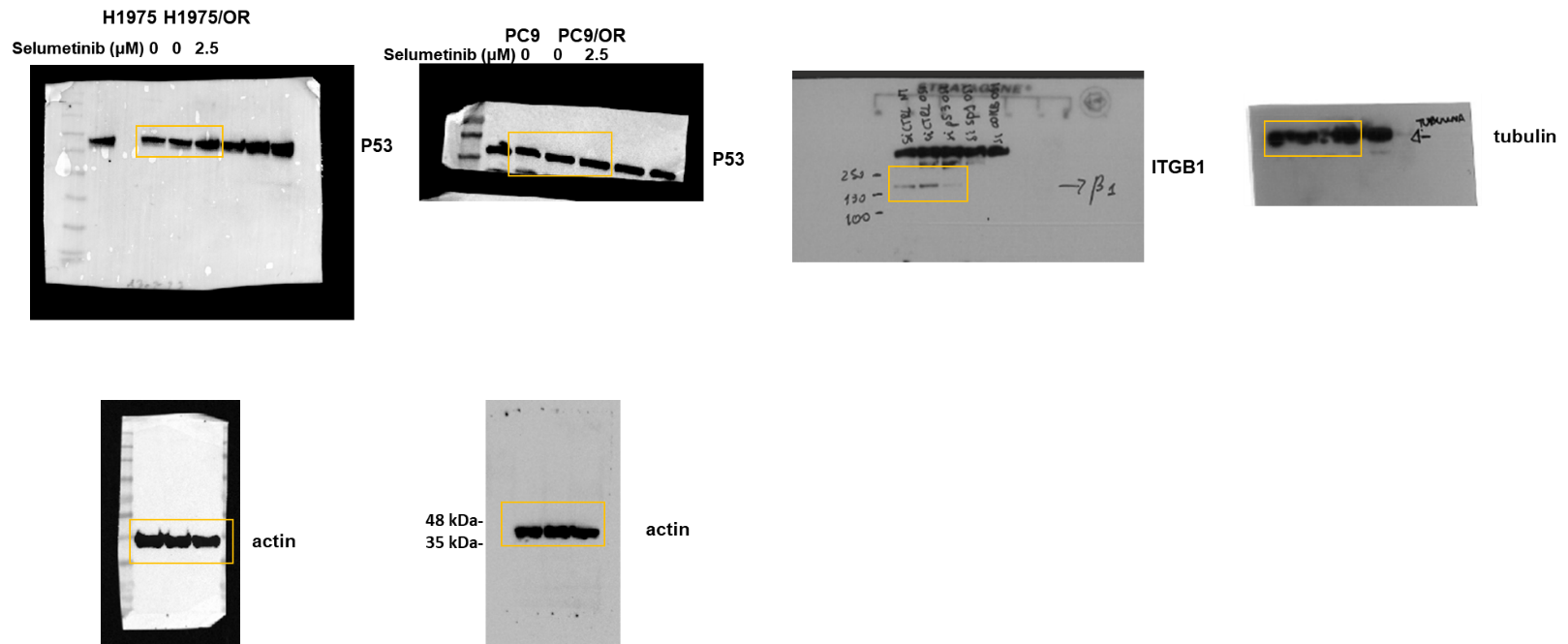
**

**Figure S12.**

**
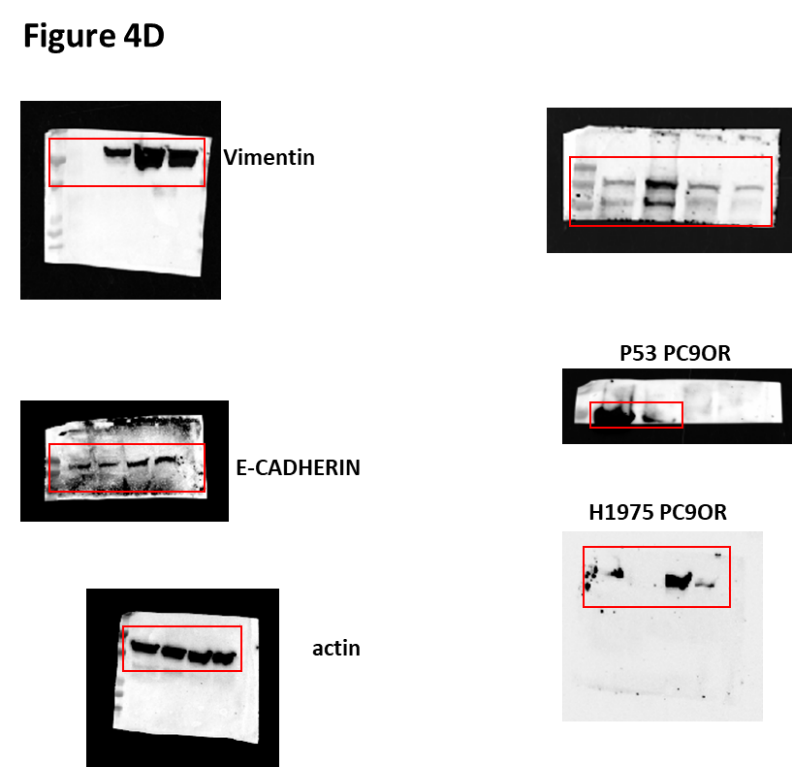
**

**Figure S13.**


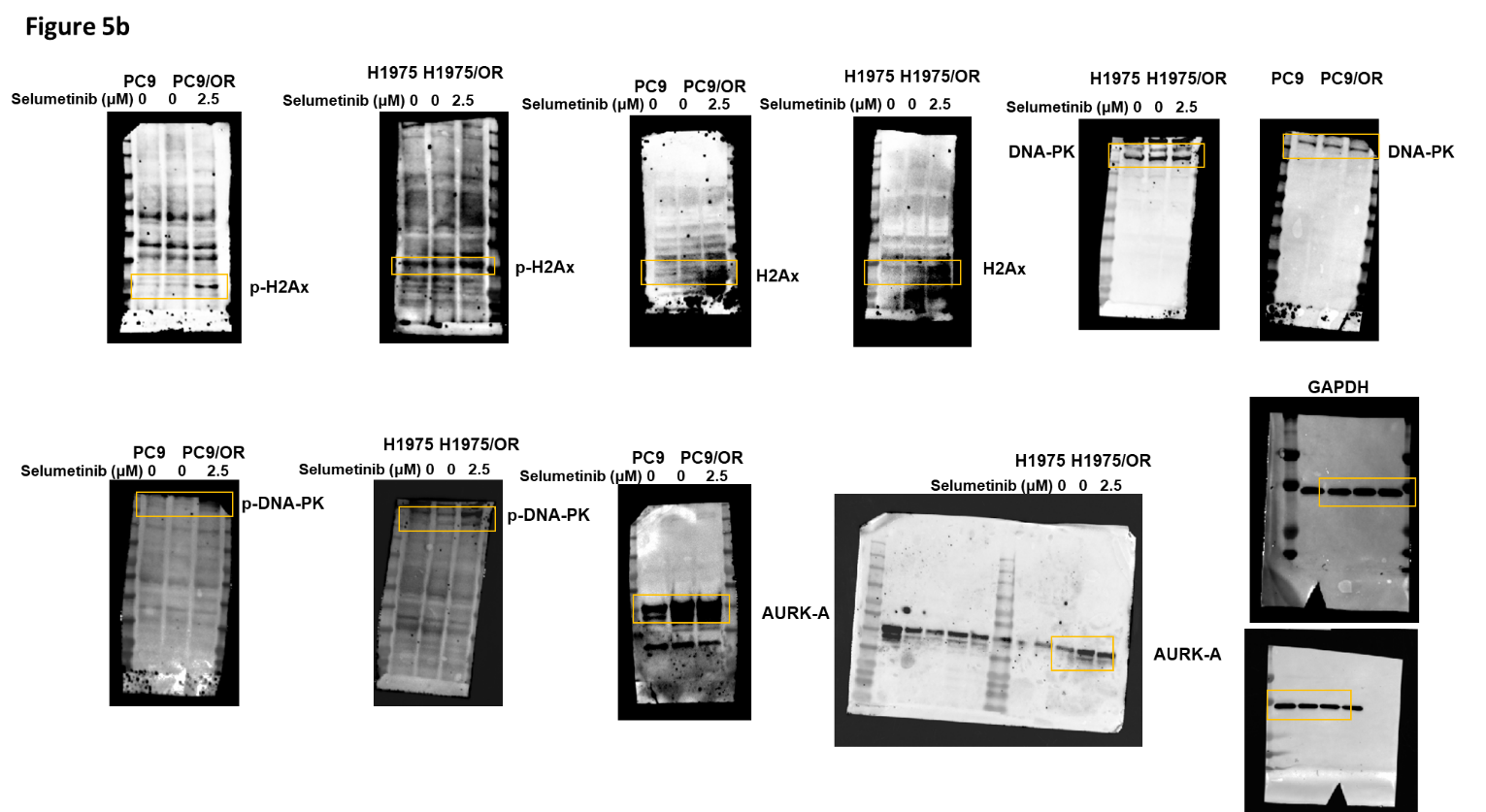


**Figure S14.**

**5c**

**
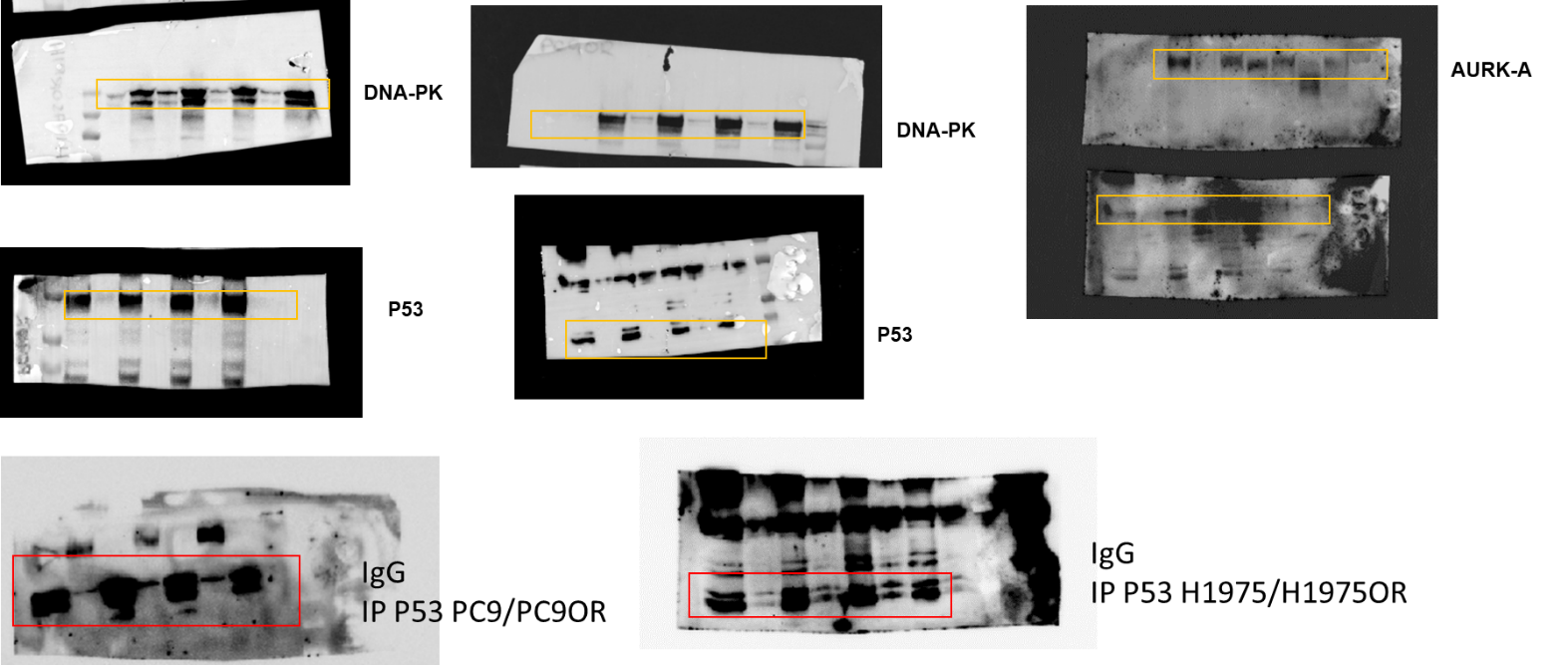
**

**Figure S15.**

**
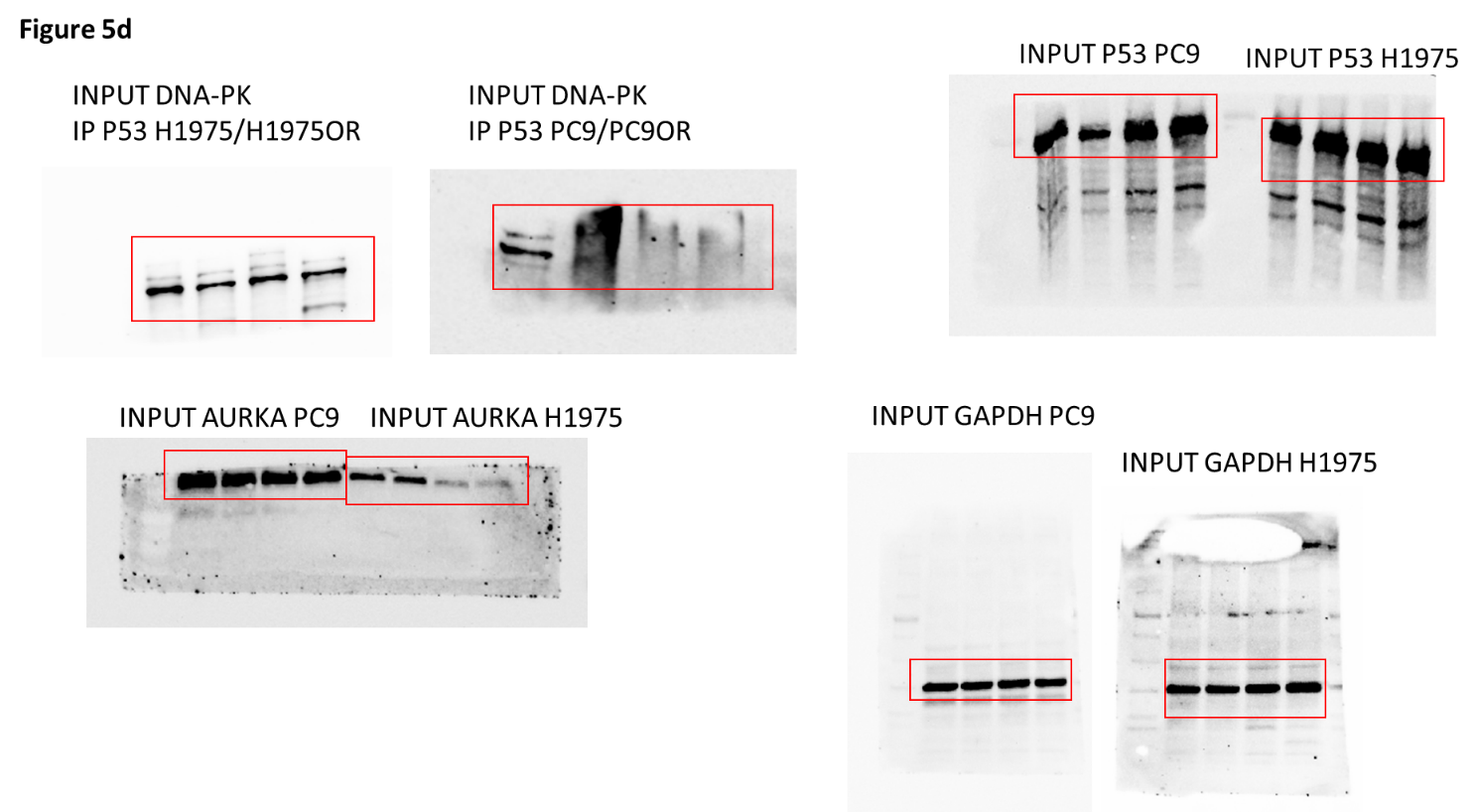
**

**Figure S16.**


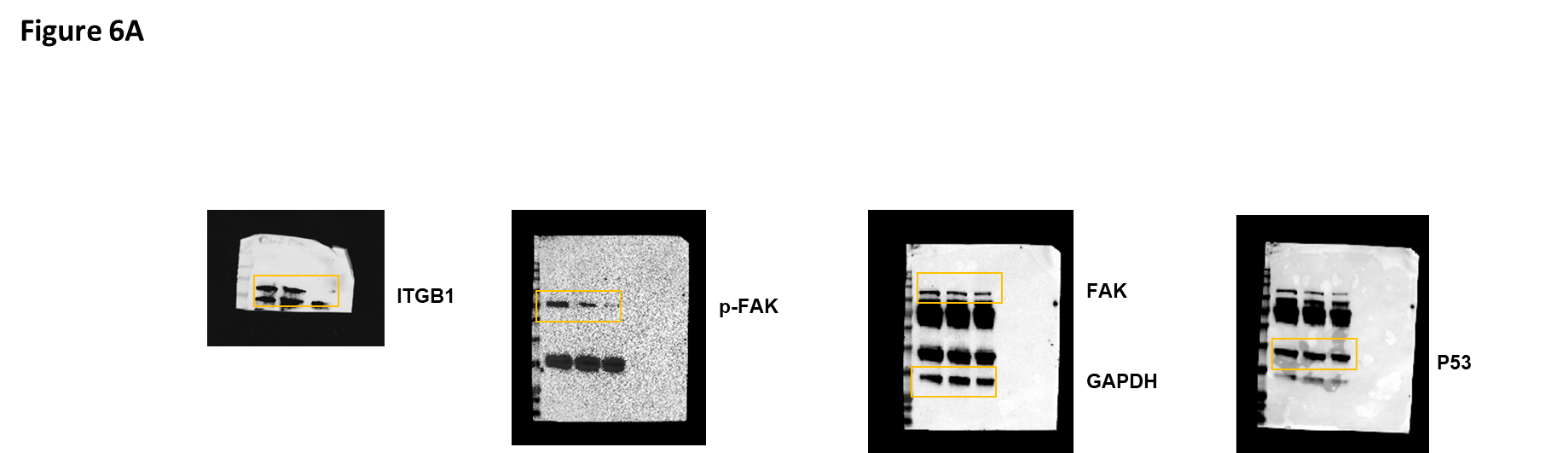


**Figure S17.**

**
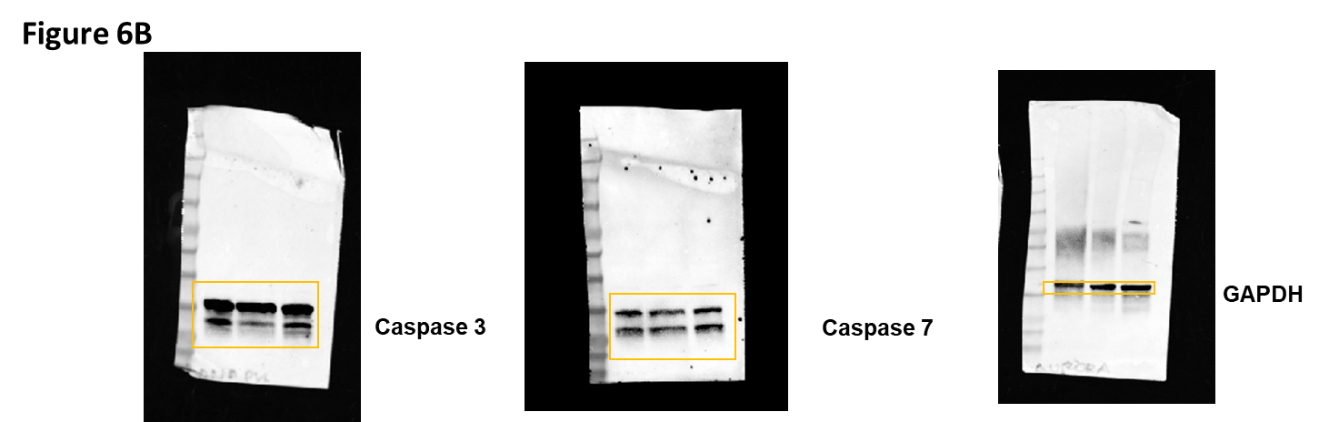
**

**
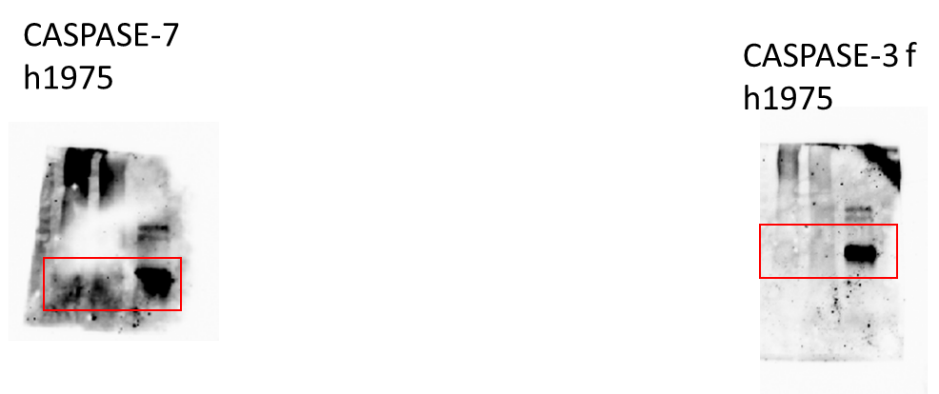
**

**Figure S18.**

**
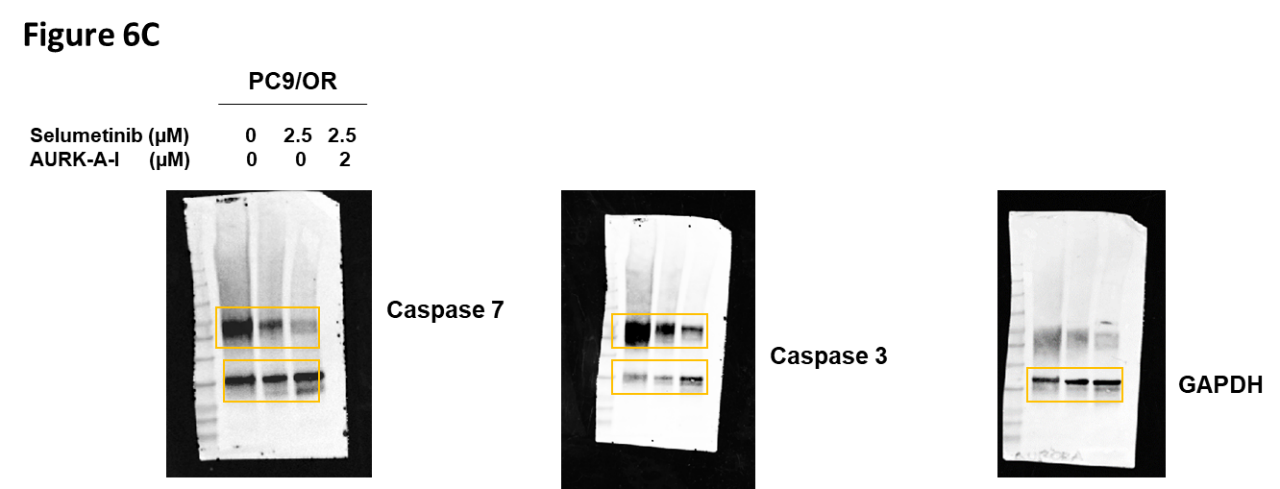
**

**Figure S19.**


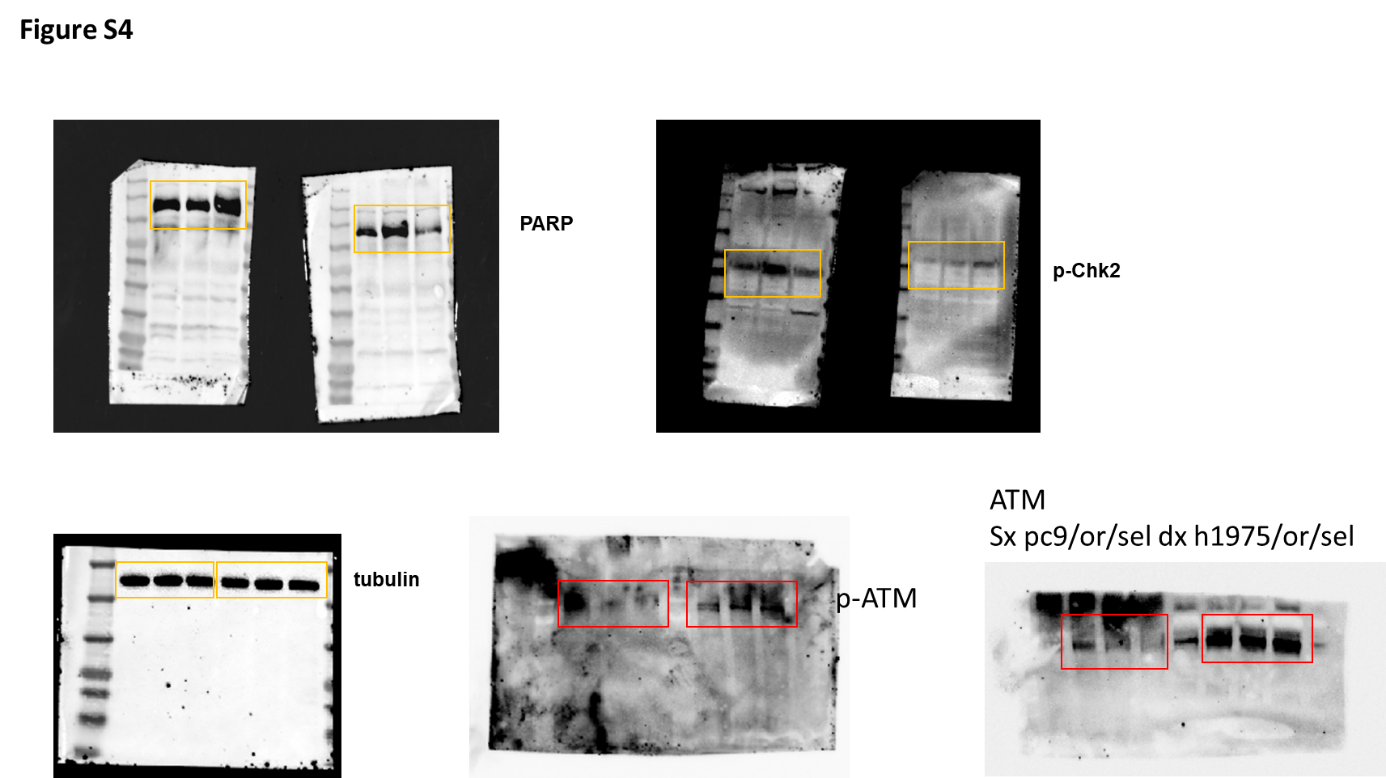


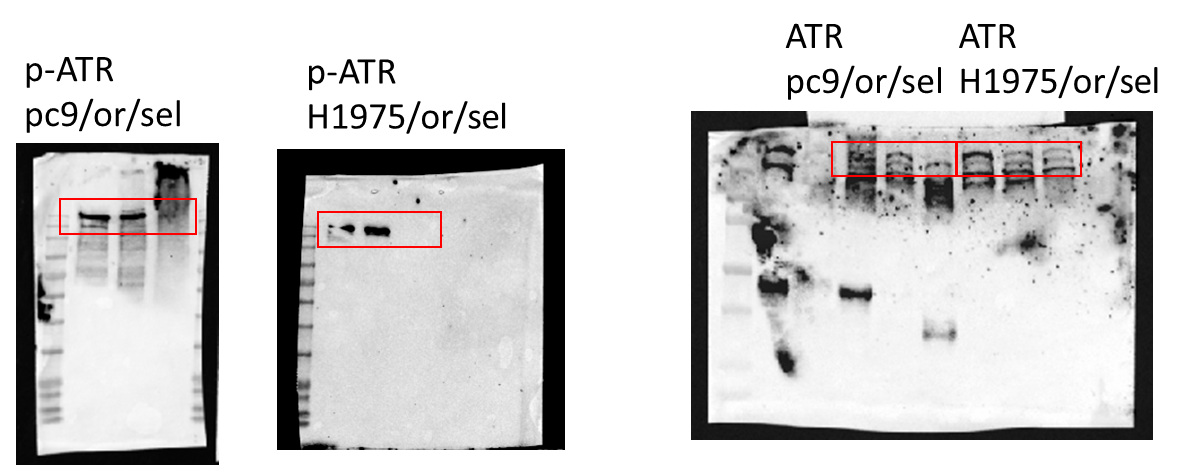

Supplement: Supplementary file 1 — Supplementary Information. [file 41598_2023_50568_MOESM1_ESM.docx]
